# Synthesis and In Vitro Antiprotozoan Evaluation of 4-/8-Aminoquinoline-based Lactams and Tetrazoles

**DOI:** 10.3390/molecules25245941

**Published:** 2020-12-15

**Authors:** Matshawandile Tukulula, Stefan Louw, Mathew Njoroge, Kelly Chibale

**Affiliations:** 1 School of Chemistry and Physics, University of KwaZulu Natal, Durban 4000, South Africa; 2 Department of Chemistry, University of Cape Town, Rondebosch 7701, South Africa; slouw@unam.na (S.L.); MN.Njoroge@uct.ac.za (M.N.); kelly.chibale@uct.ac.za (K.C.); 3 Department of Chemistry and Biochemistry, University of Namibia, Windhoek 10005, Namibia; 4 Institute of Infectious Disease and Molecular Medicine, University of Cape Town, Rondebosch 7701, South Africa; 5 South African Medical Research Council Drug Discovery and Development Research Unit, Department of Chemistry University of Cape Town, Rondebosch 7701, South Africa

**Keywords:** human African trypanosomiasis (HAT), *Plasmodium falciparum* chloroquine multidrug-resistant (K1) strain, 4-aminoquinoline β-lactams, 8-aminoquinoline tetrazoles, aqueous kinetic solubility

## Abstract

A second generation of 4-aminoquinoline- and 8-aminoquinoline-based tetrazoles and lactams were synthesized via the Staudinger and Ugi multicomponent reactions. These compounds were subsequently evaluated in vitro for their potential antiplasmodium activity against a multidrug-resistant K1 strain and for their antitrypanosomal activity against a cultured *T. b. rhodesiense* STIB900 strain. Several of these compounds (**4a**–**g**) displayed good antiplasmodium activities (IC_50_ = 0.20–0.62 µM) that were comparable to the reference drugs, while their antitrypanosomal activity was moderate (<20 µM). Compound **4e** was 2-fold more active than primaquine and was also the most active (IC_50_ = 7.01 µM) against *T. b. rhodesiense* and also exhibited excellent aqueous solubility (>200 µM) at pH 7.

## 1. Introduction

Neglected tropical and infectious diseases caused by protozoan organisms such as kinetoplastid and plasmodium parasites pose a great danger to human lives. Key among these protozoan infections are the human African trypanosomiasis (HAT), also known as sleeping sickness, caused by two *Trypanosoma brucei* subspecies (i.e., *Trypanosoma brucei rhodesiense* (*T. b. rhodesiense*) and *Trypanosoma brucie gambiense* (*T. brucie gambiense*)) and malaria caused by five plasmodium species, with *Plasmodium falciparum (P. falciparum)* being the most virulent [1,2,3,4]. HAT is fatal if left untreated, and *T. b. gambiense* accounts for about 95–97% of all cases while *T. b. rhodesiense* accounts for only 3–5% [5]. In 2018 HAT and malaria had over 229 million reported cases and 405,000 deaths, the majority being attributed to the latter [6,7]. However, the number of reported cases and deaths have been dropping significantly due, in part, to the World Health Organization (WHO) goals of eliminating both HAT and malaria [8,9].

Currently, there are no vaccines against HAT and malaria, even though both have been around for over a century. There are very few drugs available for treating HAT, and their use depends on the causative species and the stage, the disease [2,10]. Furthermore, the drawbacks of drugs such as melarsoprol, pentamidine, suramin and their combinations are the need to be administered intravenously (IV), toxicity and the ever-present threat of drug resistance emerging [11,12,13,14]. Thus, there is an urgent need for the development of a pipeline of new drugs against this disease. On the other hand, resistant forms of the plasmodium parasite are threatening the mainstay of malaria chemotherapy, artemisinin [15,16,17,18]. Therefore, there is also an urgent need for continuously developing newer drugs that will be efficacious against all forms of the plasmodia parasite.

**Figure 1:**
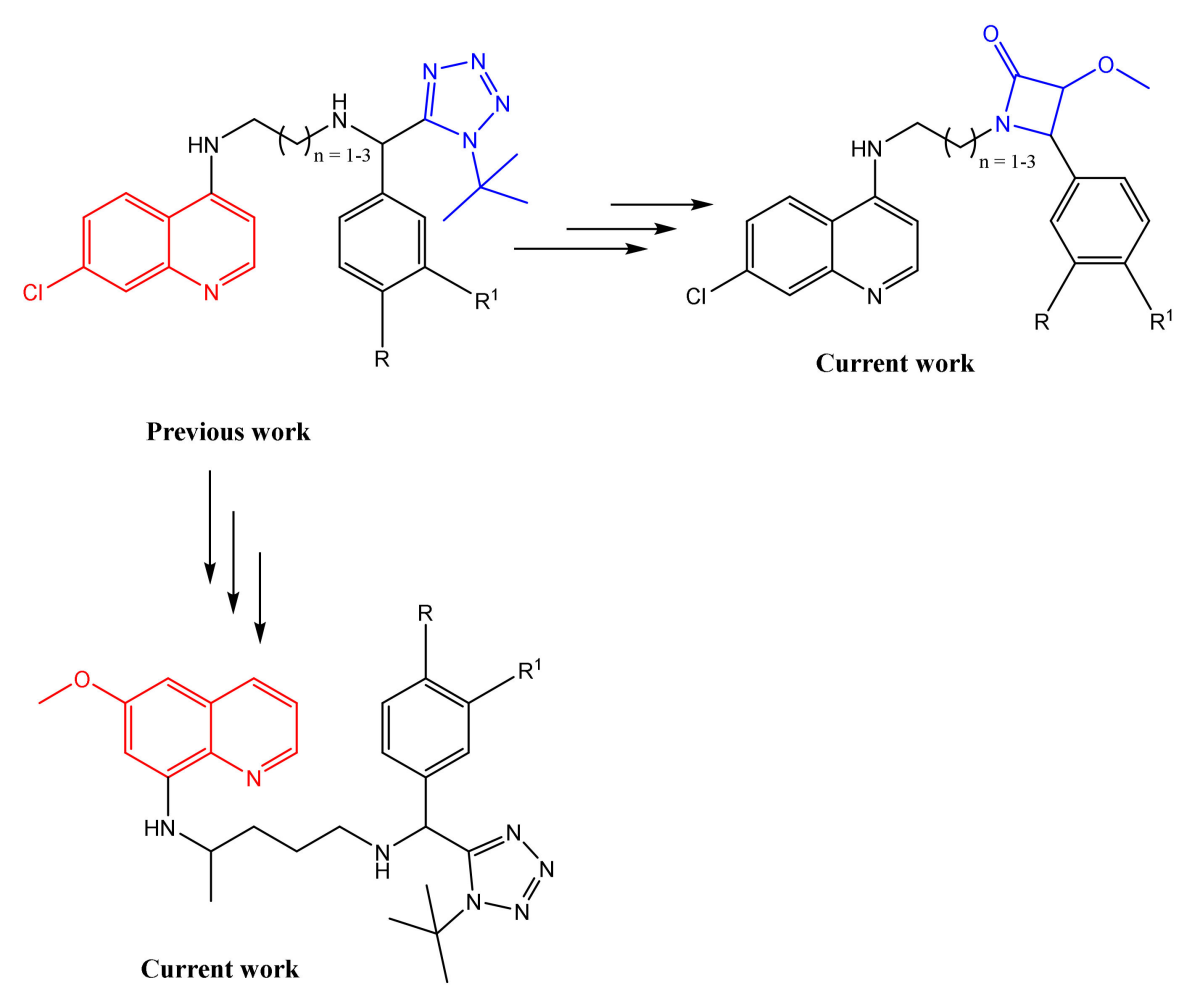
Rationale for the current work.

Previously we reported a series of 4-aminoquinoline- based tetrazole compounds that were more efficacious, against both the chloroquine (CQ)-sensitive and -resistant strains, than the reference drug CQ [19,20]. Herein we report a limited and focused number of the second generation of compounds where the tetrazole moiety is replaced by a β-lactam ring and the 4-amino-7chloroquinoline ring is replaced by primaquine, an 8-aminoquinoline (Figure 1).

In the last decade and a half, a number of literature reports have highlighted the use of the β-lactam moiety in antiprotozoal research, especially as antiplasmodium agents [21,22,23,24,25,26,27]. D’hooge et al. reported a series of nontoxic *cis*-β-lactams that exhibited IC_50_ values in the micromolar range against the CQ-sensitive D10 strain [22] and Singh et al. also reported a series of β-lactams that exhibited low micromolar potency against three *P. falciparum* strains (D10, K1 and W2) [23]. On the other hand, Kumar and coworkers also reported a number of 4-amino-7chloroquinoline-β-lactam hybrids that possessed antiplasmodium activity in the low micromolar to nanomolar range against the CQ-resistant W2 strain [24,25]. These hybrids were synthesized by coupling the 4-amino-7chloroquinoline moieties to separately prepared β-lactams. In our current work, the 4-amino-7chloroquinoline-β-lactam hybrids are prepared via the Staudinger reaction [22,28], where the 4-amino-7chloroquinoline plays the role of an amine input in the reaction. Our group also previously reported the antitrypanosomal activity of gamma- and delta-lactams [29], and to our knowledge, there has not been any literature report of the evaluation of β-lactam-quinoline hybrids as antitrypanosomal agents. This is in view of the fact that antimalarial 8-aminoquinolines, i.e., primaquine and tafenoquine, have been reported to show promising antitrypanosomal activity [30,31,32].

## 2. Results and Discussion

### 2.1. Chemistry

**Scheme 1:**
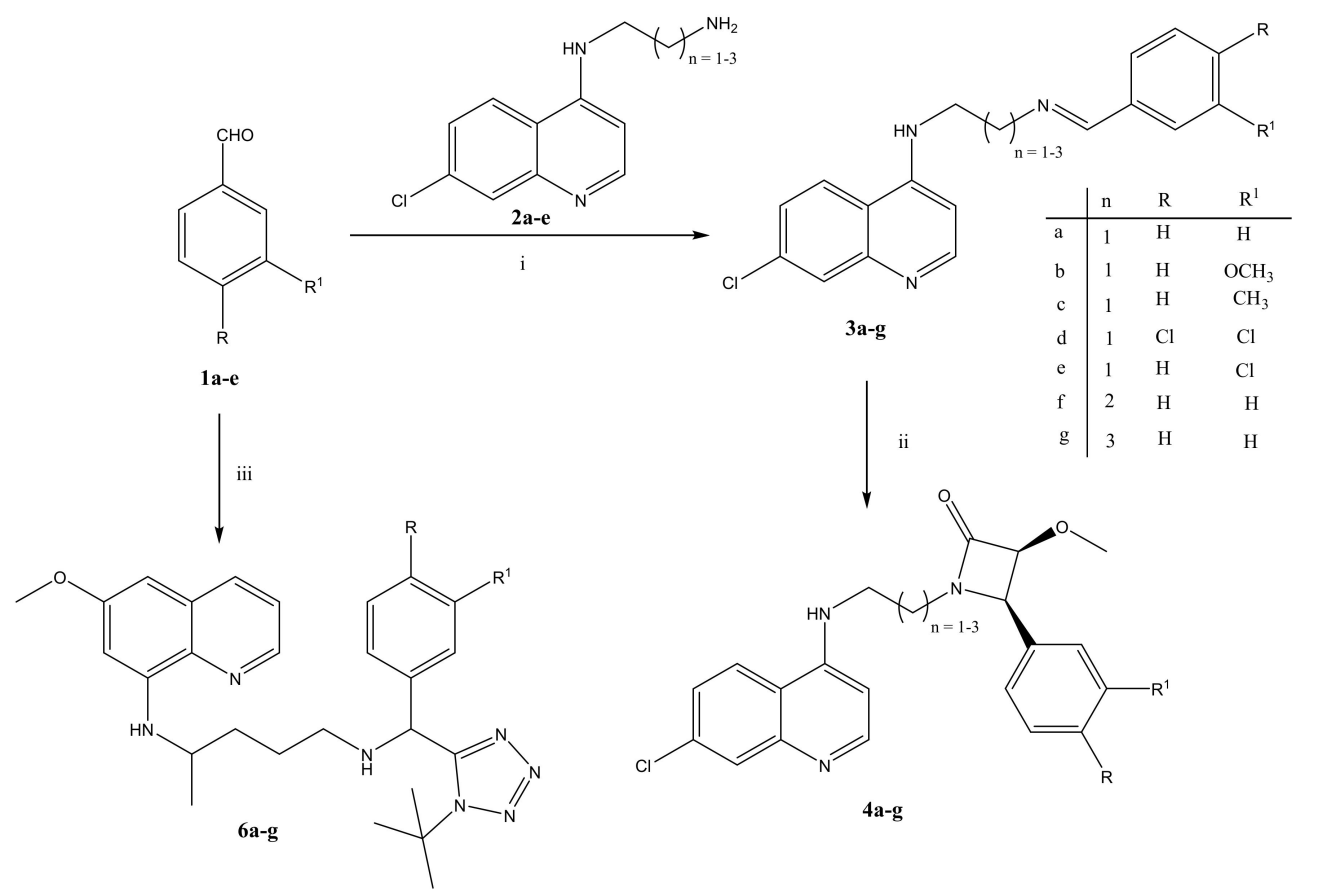
*Reagents and conditions*: (i) MeOH, rt, 24 h; (ii) methoxy acetyl chloride, Et_3_N, DCM, N_2_, −78 °C (0.5 h), then overnight for 18 h at room temperature; (iii) Primaquine diphosphate salt (**4**), Et_3_N, TMSN_3_, *t*-Butyl isocyanide, MeOH, 40 °C, 24 h.

The synthesis of the 4-amino-7chloroquinoline tethered β-lactams commenced by reacting key diamines **2a**–**c** [33] with various aromatic aldehydes (**1a**–**e**) to give Schiff-bases **3a**–**g,** which were dried under vacuum prior to use in the next step without any further purification (Scheme 1). The Staudinger reaction of the Schiff-bases with the methoxy acetyl chloride exclusively furnished the *cis* β-lactam products (**4a**–**g**) in 4–30% yields and over 90% purity except for **4f** that still contained starting Schiff base traces even after extensive purification. The *cis* configuration was assigned based on the ^1^H NMR model proposed by Xu and coworkers [34,35]. According to the model, when the *J*-coupling constant between the two protons on the adjacent chiral carbons of the β-lactam ring is between 4 and 6 ppm, then the configuration is *cis*, while between 2 and 3 ppm indicates *trans*. The same aromatic aldehydes (**1a**–**e**) used in β-lactams synthesis were also subjected to the isocyanide-based Ugi multicomponent reaction involving TMSN_3_ and commercially available primaquine diphosphate salt (**5**) following the method as reported by our group [19,36,37,38] (Scheme 1). The desired target compounds (**6a**–**e**) were obtained in 21–50% yields as 1:1 diastereomeric mixtures due to the fact that the primaquine salt used in this study is racemic. Attempts to separate/resolve the diastereomeric mixture using HPLC was unsuccessful owing to the fact these diastereomeric mixtures coalesce into a single sharp peak in the HPLC chromatograms (Figures S1–S5) irrespective of whether the gradient of the mobile phase or pH is varied. Thus, for subsequent biological studies, these 1:1 diastereomeric mixtures were used without being separated/resolved. All the synthesized target compounds were fully characterized using routine spectroscopic methods (NMR, IR, MS) (see Table S1 and NMR Figures in supplementary material) NMR, IR, MS) and their yields and HPLC purities can be found in Table S1 in the supplementary material.

### 2.2. In Vitro Antiprotozoan Activities

All the synthesized compounds were evaluated in vitro for their antiplasmodium (against the multidrug K1 resistant strain) and antitrypanosomal activity (against the cultured *T. b. brucei rhodesiense* STIB900 parasite strain). Chloroquine, primaquine, melarsoprol and podophyllotoxin were used as a positive control, and the results are tabulated in Table 1. Generally, all β-lactam based compounds were more potent than primaquine-based tetrazoles. Furthermore, all the β-Lactam based compounds also showed superior antiplasmodium activity compared to primaquine, with the most active 3,4-*dichloro* substituted **4d** (IC_50_ = 0.20 µM) being 3-fold more active than primaquine and equipotent to chloroquine. The β-lactam based compounds with the butyl spacer (IC_50_ = 0.31 µM) was the most favored compound to the ones with the ethyl (IC_50_ = 0.47 µM) and propyl spacers (IC_50_ = 0.57 µM). None of the primaquine-based tetrazoles had better antiplasmodium activity than the two reference drugs, with the most active, 3,4-*dichloro* substituted **6d** (IC_50_ = 1.31 µM), being 2-fold less active than primaquine and 6-fold less active than chloroquine. In terms of electronic effects, compounds with an aromatic ring containing moderately electron-withdrawing groups (EWG) (Cl or diCl) substitutions fared better than those contain electron-donating groups (EDG) (Me and OCH_3_) in both sets of compounds. The selective indices of some of the active β-lactam-based compounds were closer to or greater than 100, indicating that these compounds are more selective towards the chloroquine-resistant *Plasmodium falciparum* parasite. Disappointingly, none of these compounds were as potent as the first-generation compounds against the K1 strain.

Against *T. b. brucei rhodesiense*, all the tested compounds showed moderate activity, and none of them had superior activity than the reference drug, melarsoprol. Compounds **4e** (IC_50_ = 7.01 µM) and **6b** (IC_50_ = 14.16 µM) were the most active in the β-lactam and tetrazole series, respectively. Unlike in the case of activity against *Plasmodium falciparum*, the compound with the propyl spacer (IC_50_ = 16.09 µM) was more active than compounds with ethyl (IC_50_ = 53.16 µM) and butyl spacers (IC_50_ = 20.00 µM). In terms of electronic effects, compounds containing EWG in the β-lactam series were more active than those containing EDG. However, in the tetrazole series, no electronic effect trend could be delineated since all the compounds had comparable activities.

### 2.3. Solubility of Selected Compounds

A limited number of compounds from each series were selected for assessment of their aqueous kinetic solubility at pH 7.0:**4a**, **4e** and **4g** from the β-lactam series and **6c**–**e** from the tetrazole series (Table 2). These compounds were selected based on their good antiplasmodium (**4a**, **4e** and **4g**), promising antitrypanosomal (**4d** and **6c**–**e**) and excellent cytotoxicity (**4a** and **6d**) profiles. The β-lactam series of compounds generally exhibited excellent solubility (>200 µM) compared to the tetrazole series compounds (<20 µM).

## 3. Materials and Methods

### 3.1. General Information

All chemical reagents used were supplied by either Sigma-Aldrich^®^ (Johannesburg, South Africa) or Merck (Johannesburg, South Africa) and were used without further purification. Unless otherwise stated, all the solvents used were anhydrous and were purchased from Sigma-Aldrich, with the exception of THF and diethyl ether, which were dried in-house prior to use. Chromatographic solvents such as Ethyl acetate, dichloromethane and Hexane were purchased from Kimix (Cape Town, South Africa) or Protea Chemicals (Cape Town, South Africa) and were distilled prior to use. HPLC grade methanol, acetonitrile and formic acid (98–100%) were purchased from Sigma-Aldrich. Ultrafiltered water was purified by a Millipore Synergy water purification system (Microsep, Tygervalley, South Africa) and was used as such. The progress of the reactions was monitored by thin-layer chromatography (TLC) using Merck PF_254_ aluminum-backed precoated silica gel plates and viewed under ultraviolet (UV) light, and where necessary, visualized using iodine vapor. Product purification on the flash column chromatography was carried out using Merck Kieselgel 60:70–230 mesh. Both analytical and preparative HPLC were performed on a modular Waters HPLC system consisting of a 2767 sample manager, 2545 quaternary gradient pump, 1500 series column heater and a 2998 photodiode array detector (PDA) with a Prep 2998 flow-cell. Waters Xbridge C_18_, 4.6 × 150 mm column with 5 μm particles was used for the analytical scale HPLC analysis and a Waters Xbridge C_18_, OBD 19 × 250 mm preparative HPLC column was used for the purifications. Xbridge C_18_, 5 μm, 4.6 × 20 mm and Xbridge C_18_, 5 μm, 19 × 10 mm guard columns were connected to the inlets of the analytical and preparative HPLC columns, respectively (Microsep, Tygervalley, South Africa).

Melting points were determined on a Reichert-Jung Thermovar hot-stage microscope and are uncorrected. Mass spectra were obtained by flow-injection (5 mM NH_4_ formate pH 3 in H_2_O:ACN, no column used) on an AB SCIEX 4000 QTRAP Hybrid triple quadrupole linear ion trap mass spectrometer, coupled with an Agilent 1200 Rapid Resolution (600 bar) HPLC system consisting of a binary pump, degasser, autosampler and temperature-controlled column compartment (SCIEX, Johannesburg, South Africa). Infra-red spectra were recorded on a PerkinElmer Spectrum 100 FT-IR spectrometer (Perkin Elmer SA, Midrand, South Africa) in the 4000–450 cm^−1^ range, with samples dissolved in dichloromethane solution or placed in KBr discs. NMR spectra were recorded on Bruker 400 MHz (Bruker, Bremen, Germany) and/or Varian Unity 400 MHz and/or Varian Mercury 300 MHz spectrometers (Agilent Technologies, CA, USA). All chemical shifts are reported in ppm and were referenced using solvent signals (2.50 and 39.4 ppm for DMSO-*d*_6_ and 7.6 and 77.0 ppm for CDCl_3_). Chemical shifts (δ) are recorded in parts per million (ppm). Coupling constants, *J*, are measured in Hertz (Hz) and rounded off to one decimal place. Abbreviations used in the assignment of the ^1^H NMR spectra are as follows: br (broad), d (doublet), dd (doublet of doublets), m (multiplets), s (singlet) and t (triplet). ^13^C NMR chemical shifts are listed without assignment to specific carbon atoms.

### 3.2. Synthetic Procedures

#### 3.2.1. General Procedure for the β-Lactam Based Compounds

Quinoline diamine **2a**–**c** (1.0 mmol) and various aldehydes **1a**–**e** (1.0 mmol) were stirred in anhydrous methanol at room temperature for 24 h to afford Schiff-based **3a**–**g** that were subsequently used, after drying in vacuum for 4 h, in the next step without any further purification. Dry DCM (5 mL), followed by Et_3_N (3.0 eq), was added to the Schiff-based (1.0 eq), and the resulting mixtures were stirred for 30 min at −78 °C under an inert N_2_ atmosphere. Thereafter, methoxy acetyl chloride (1.5 eq) in dry DCM (2 mL) was added to the mixtures dropwise, mixtures allowed to warm to room temperature and further stirred overnight for 18 h at room temperature. On completion, saturated NaHCO_3_ was then added to the mixtures and the organic layers extracted with DCM. The combined organic extracts were then washed with brine and water, dried over anhydrous MgSO_4_ and solvent removed in vacuo to yield crude products, which were purified by column chromatography to exclusively give the *cis* β-lactam based compounds:

*1-[2-(7-Chloroquinolin-4-ylamino)ethyl]-3-methoxy-4-phenylazetidin-2-one***4a**, as a white solid (55 mg, 30%); m. p. 142–146 °C, R_f_ (DCM: MeOH, 95: 0.5%) 0.21; IR ν_max_ (DCM)/cm^−1^ 1744 (C = O), 1620 (Ar C = C), 1264 (C-O ester); δ_H_ (400 MHz; CDCl_3_) 8.49 (1H, d, *J* 5.4 Hz, H2), 8.00 (1H, d, *J* 2.1 Hz, H8), 7.84 (1H, d, *J* 9.0 Hz, H5), 7.42 (1H, dd, *J* 9.0 and 2.1 Hz, H6), 7.35 (5H, m, ArH), 6.26 (1H, d, *J* 5.4 Hz, H3), 4.97 (1H, d, *J* 4.4 Hz, H1′), 4.74 (1H, d, *J* 4.4 Hz, H2′), 3.54 (2H, m, H9), 3.46 (2H, m, H10), 3.12 (3H, s, H6′); δ_C_ (101 MHz; CDCl_3_) 168.9, 151.5, 149.8, 148.8, 135.3, 133.2, 129.1, 128.7 (2C), 128.4, 128.3 (2C), 125.8, 121.9, 117.3, 98.5, 85.4, 63.3, 58.2, 42.4 and 41.1; LCMS *m/z* 382 [M + H]^+^, HPLC purity: 95.6%; t_r’_ = 8.37 min.

*1-[2-(7-Chloroquinolin-4-ylamino)ethyl]-3-methoxy-4-(4-methoxyphenyl)azetidin-2-one***4b**, as a yellow solid (44.6 mg, 9%); m. p. 144–147 °C, R_f_ (DCM: MeOH, 95: 0.5%) 0.21; IR ν_max_ (DCM)/cm^−1^ 1746 (C = O), 1610 (Ar C = C), 1267 (C-O ester); δ_H_ (400 MHz; CDCl_3_) 8.48 (1H, d, *J* 5.5 Hz, H2), 7.98 (1H, s, H8), 7.84 (1H, d, *J* 8.9 Hz, H5), 7.41 (1H, d, *J* 9.0, H6), 7.24 (2H, d, *J* 8.6 Hz, 2 × H4′), 6.86 (2H, d, *J* 8.6 Hz, 2 × H3′), 6.45 (1H, br s, NH), 6.25 (1H, d, *J* 5.5 Hz, H3), 4.74 (1H, d, *J* 4.4 Hz, H1′), 4.71 (1H, d, *J* 4.4 Hz, H2′), 3.79 (3H, s, H5′), 3.52 (2H, t, *J* 4.6 Hz, H9), 3.47 (1H, m, H*10a*), 3.41 (1H, m, H*10b*), 2.92 (3H, s, H6′); δ_C_ (101 MHz; CDCl_3_) 169.0, 160.3, 151.1, 150.1, 148.3, 135.5, 129.7 (2C), 127.9, 125.9, 124.8, 122.0, 117.2, 114.1 (2C), 98.4, 85.3, 62.9, 58.2, 55.3, 42.5 and 40.9; LCMS *m/z* 412 [M + H]^+^, HPLC purity: 95.6%; t_r’_ = 8.43 min.

*1-[2-(7-Chloroquinolin-4-ylamino)ethyl]-3-methoxy-4-p-tolylazetidin-2-one***4c**, as a yellow solid (21 mg, 6%); m. p. 78–82 °C, R_f_ (DCM: MeOH, 95: 0.5%) 0.31; IR ν_max_ (DCM)/cm^−1^ 1744 (C = O), 1604 (Ar C = C), 12,652 (C-O ester); δ_H_ (400 MHz; CDCl_3_) 8.49 (1H, d, *J* 1.3 Hz, H8), 8.04 (1H, d, *J* 5.7 Hz, H2), 7.88 (1H, d, *J* 9.0 Hz, H5), 7.41 (1H, dd, *J* 9.0 and 1.3 Hz, H6), 7.20 (2H, d, *J* 8.0 Hz, 2 × H4′), 7.14 (2H, d, *J* 8.0 Hz, 2 × H3′), 6.78 (1H, br s, NH), 6.24 (1H, d, *J* 5.7 Hz, H3), 4.77 (1H, d, *J* 4.3 Hz, H1′), 4.73 (1H, d, *J* 4.3 Hz, H2′), 3.52 (2H, m, H9), 3.47 (2H, m, H10), 3.13 (3H, s, H6′), 2.35 (3H, s, H5′); δ_C_ (101 MHz; CDCl_3_) 169.0, 150.7, 150.1, 147.1, 139.1, 136.0, 129.4 (2C), 128.9, 128.4 (2C), 126.8, 126.1, 122.3, 117.0, 98.2, 85.3, 63.1, 58.2, 42.5, 40.9 and 21.6; LCMS *m/z* 396 [M + H]^+^, HPLC purity: 94.6%; t_r’_ = 8.29 min.

*1-[2-(7-Chloroquinolin-4-ylamino)ethyl]-4-(3,4-dichlorophenyl)-3-methoxyazetidin-2-one***4d**, as a pale-yellow solid (17.3 g, 4%); m. p. 152–156 °C, R_f_ (DCM: MeOH, 95: 0.5%) 0.16; IR ν_max_ (DCM)/cm^−1^ 1739 (C = O), 1601 (Ar C = C), 1277 (C-O ester); δ_H_ (400 MHz; CDCl_3_) 8.45 (1H, d, *J* 5.6 Hz, H2), 7.96 (1H, d, *J* 2.1 Hz, H8), 7.89 (1H, d, *J* 9.0 Hz, H5), 7.39 (3H, m, ArH), 7.16 (1H, dd, *J* 9.0 and 2.1 Hz, H6), 6.74 (1H, br s, NH), 6.25 (1H, d, *J* 5.6 Hz, H3), 4.78 (1H, d, *J* 4.4 Hz, H1′), 4.74 (1H, d, *J* 4.4 Hz, H2′), 3.49 (2H, m, H9), 3.43 (2H, m, H10), 3.18 (3H, s, H6′); δ_C_ (101 MHz; CDCl_3_) 168.6, 150.5, 150.1, 147.2, 136.0, 133.7, 133.2, 130.6, 130.4, 127.6, 127.0, 126.2, 126.1, 122.2, 117.0, 98.3, 85.5, 62.1, 58.6, 42.2 and 41.0; LCMS *m/z* 450 [M + H]^+^, HPLC purity: 99.5%; t_r’_ = 8.81 min.

*1-[2-(7-Chloroquinolin-4-ylamino)ethyl]-4-(4-chlorophenyl)-3-methoxyazetidin-2-one***4e**, as a pale-yellow solid (20.3 mg, 14%); m. p. 135–138 °C, R_f_ (DCM: MeOH, 95: 0.5%) 0.14; IR ν_max_ (DCM)/cm^−1^ 1740 (C = O), 1598 (Ar C = C), 1265 (C-O ester); δ_H_ (400 MHz; CDCl_3_) 8.46 (1H, d, *J* 5.3 Hz, H2), 7.95 (1H, d, *J* 1.9 Hz, H8), 7.85 (1H, d, *J* 9.0 Hz, H5), 7.39 (1H, dd, *J* 9.0 and 1.9 Hz, H6), 7.31 (2H, d, *J* 8.5 Hz, 2 × H4′), 7.24 (2H, d, *J* 8.5 Hz, 2 × H3′), 6.47 (1H, br s, NH), 6.24 (1H, d, *J* 5.3 Hz, H3), 4.77 (1H, d, *J* 4.3 Hz, H1′), 4.72 (1H, d, *J* 4.3 Hz, H2′), 3.54 (1H, m, H*10a*), 3.47 (3H, m, H9 and H*10b*), 3.13 (3H, s, H6′); δ_C_ (101 MHz; CDCl_3_) 168.7, 151.2, 150.0, 148.4, 135.4, 135.1, 131.2, 129.7 (2C), 128.9 (2C), 128.0, 125.9, 122.0, 117.2, 98.5, 85.4, 62.6, 58.3, 42.3 and 41.0; LCMS *m/z* 416 [M + H]^+^, HPLC purity: 99.2%; t_r’_ = 8.63 min.

*1-[3-(7-Chloroquinolin-4-ylamino)propyl]-3-methoxy-4-phenylazetidin-2-one***4f**, as a yellow paste (19.6 mg, 5%); R_f_ (DCM: MeOH, 95: 0.5%) 0.41; IR ν_max_ (DCM)/cm^−1^ 1745 (C = O), 1609 (Ar C = C), 1269 (C-O ester); δ_H_ (300 MHz; CDCl_3_) 8.59 (1H, d, *J* 5.4 Hz, H2), 8.11–8.13 (2H, m, H5 and H8), 7.99 (1H, d, *J* 9.0 Hz, H6), 7.47–7.52 (5H, m, ArH), 6.51 (1H, br s, NH), 6.46 (1H, d, *J* 5.4 Hz, H3), 4.85 (1H, d, *J* 4.4 Hz, H1′), 4.81 (1H, d, *J* 4.4 Hz, H2′), 3.60 (3H, s, H6′), 3.49 (2H, m, H11), 3.28 (2H, m, H9), 1.97 (2H, m, H10; δ_C_ (75 MHz; CDCl_3_) 169.2, 155.2, 147.5, 140.1, 136.8, 136.2, 129.0, 128.7, 128.5, 128.1, 126.6, 125.9, 124.3, 122.1, 117.5, 98.5, 85.1, 61.9, 57.8, 42.1, 40.5 and 26.7; LCMS *m/z* 397 [M + H]^+^.

*1-[4-(7-Chloroquinolin-4-ylamino)butyl]-3-methoxy-4-phenylazetidin-2-one***4g**, as a yellow solid (28 mg, 9%); m. p. 86–89 °C, R_f_ (DCM: MeOH, 95: 0.5%) 0.31; IR ν_max_ (DCM)/cm^−1^ 1739 (C = O), 1599 (Ar C = C), 1258 (C-O ester); δ_H_ (400 MHz; CDCl_3_) 8.39 (1H, d, *J* 9.0 Hz, H5), 8.28 (1H, d, *J* 5.6 Hz, H2), 8.05 (1H, s, H8), 7.92 (1H, br s, NH), 7.36 (6H, m, H6 and ArH), 6.39 (1H, d, *J* 5.6 Hz, H3), 4.76 (1H, d, *J* 4.3 Hz, H1′), 4.73 (1H, d, *J* 4.3 Hz, H2′), 3.44 (4H, m, H9 and H12), 3.09 (3H, s, H6′), 1.92 (2H, m, H10), 1.72 (2H, m, H11); δ_C_ (101 MHz; CDCl_3_) 167.7, 153.5, 145.6, 142.7, 137.7, 133.5, 128.8, 128.5 (2C), 128.4, 126.7, 126.2, 124.1, 123.1, 116.3, 98.1, 85.4, 62.4, 58.1, 43.2, 40.9, 25.6 and 25.1; LCMS *m/z* 411 [M + H]^+^, HPLC purity: 98.0%; t_r’_ = 9.12 min.

#### 3.2.2. General Procedure for the Tetrazole-Based Compounds

The procedure reported previously by Tukulula et al. [19,36,37,38] was followed. Briefly, the commercially available racemic primaquine diphosphate salt **4** (1.1 mmol) was reacted, in Ugi multicomponent reaction conditions, with aromatic aldehydes **1a**–**e** (1.1 mmol), TMSN_3_ (1.1 mmol) and tert-butyl isocyanide (1.1 mmol) in the presence of triethylamine (4.0 eq) at 40 °C for 24 h to afford crude desired compounds as unresolved racemic 1:1 diastereomeric mixtures **6a**–**e**. These the diastereomeric mixtures were then purified by both column chromatography and by HPLC with no success in resolving/separating them:

*N-{5-[(1-tert-butyl-1H-tetrazol-5-yl)(phenyl)methylamino]pentan-2-yl}-6-methoxyquinolin-8-amine***6a**, as a thick yellow oil (108.4 mg, 21%); R_f_ (DCM: acetone, 95: 05%) 0.50; IR ν_max_ (DCM)/cm^−1^ 1615 (Ar C = C), 1385 (N = N), 1315 (C = N), 1266 (C-N), 1254 (C-O ester); δ_H_ (300 MHz; CDCl_3_) 8.47 (1H, m, H2), 7.88 (1H, dd, *J* 8.3 and 1.6 Hz, H4), 7.25 (6H, m, ArH and H3), 6.29 (1H, d, *J* 2.3 Hz, H7), 6.23 (1H, d, *J* 2.5 Hz, H5), 5.99 (1H, t, *J* 8.6 Hz, NH), 5.22 (1H, d, *J* 6.7 Hz, H14), 3.85 (3H, s, OCH_3_), 3.56 (1H, m, H9), 2.53 (2H, m, H13), 1.63 (4H, m, H11 and H12), 1.55 (9H, d, *J* 4.04 Hz, 3 × H18), 1.25 (3H, d, *J* 6.4 Hz, H10); δ_C_ (75 MHz; CDCl_3_) 159.5, 155.7, 145.0, 144.2, 138.9, 135.4, 134.7, 129.9, 128.9 (2C), 128.2, 128.0 (2C), 121.8, 96.6, 91.6, 61.2, 59.0, 55.2, 55.1, 47.9, 34.2, 30.0 (3C), 26.5 and 20.5; LCMS *m/z* 474 [M + H]^+^; HPLC purity: 99.3%; t_r’_ = 16.53 min.

*N-{5-[(1-tert-butyl-1H-tetrazol-5-yl)(methoxyphenyl)methylamino]pentan-2-yl}-6-methoxyquinolin-8-amine***6b**, as a yellow oil (219.6 mg, 40%); R_f_ (DCM: acetone, 95: 05%) 0.54; IR ν_max_ (DCM)/cm^−1^ 1615 (Ar C = C), 1387 (N = N), 1269 (C = N), 1266 (C-N), 1217 (C-O ester); δ_H_ (400 MHz; CDCl_3_) 8.50 (1H, m, H2), 7.91 (1H, dd, *J* 8.3 and 1.8 Hz, H4), 7.29 (1H, dd, *J* 8.2 and 4.2 Hz, H3), 7.18 (2H, m, 2 × H16), 6.83 (2H, m, 2 × H15), 6.32 (1H, d, *J* 1.7 Hz, H7), 6.26 (1H, d, *J* 1.7 Hz, H5), 6.03 (1H, m, NH), 5.20 (1H, d, *J* 9.0 Hz, H14), 3.85 (3H, d, *J* 1.4 Hz, OCH_3_), 3.77 (3H, d, *J* 1.0 Hz, H17), 3.61 (1H, m, H9), 2.54 (2H, m, H13), 1.67 (4H, m, H11 and H12), 1.57 (9H, d, *J* 5.3 Hz, 3 × H18), 1.28 (3H, d, *J* 6.4 Hz, H10); δ_C_ (101 MHz; CDCl_3_) 159.4, 155.9, 145.1, 144.2 (2C), 135.4, 134.7 (2C), 131.0, 129.9, 129.2 (2C), 121.8, 114.3 (2C), 96.6, 91.6, 61.1, 58.3, 55.2, 47.9 (2C), 34.2, 30.0 (3C), 26.5 and 20.5; LCMS *m/z* 505 [M + H]^+^; HPLC purity: 99.5%; t_r’_ = 16.39 min.

*N-{5-[(1-tert-butyl-1H-tetrazol-5-yl)(p-tolyl)methylamino]pentan-2-yl}-6-methoxyquinolin-8-amine***6c**, as a thick yellow oil (265.9 mg, 50%); R_f_ (DCM: acetone, 95: 05%) 0.51; IR ν_max_ (DCM)/cm^−1^ 1617 (Ar C = C), 1390 (N = N), 1325 (C = N), 1260 (C-N), 1232 (C-O ester); δ_H_ (400 MHz; CDCl_3_) 8.53 (1H, m, H2), 7.93 (1H, dd, *J* 8.3 and 1.7 Hz, H4), 7.31 (1H, dd, *J* 8.3 and 4.2 Hz, H3), 7.12–7.18 (4H, m, 2 × H16 and 2 × H15), 6.34 (1H, d, *J* 2.4 Hz, H7), 6.28 (1H, d, *J* 2.5 Hz, H5), 6.04 (1H, m, NH), 5.29 (1H, d, *J* 8.2 Hz, H14), 3.91 (3H, d, *J* 1.3 Hz, OCH_3_), 3.62 (1H, m, H9), 2.57 (2H, m, H13), 2.33 (3H, s, H17), 1.69 (4H, m, H11 and H12), 1.60 (9H, d, *J* 5.4 Hz, 3 × H18), 1.25 (3H, d, *J* 6.4 Hz, H10); δ_C_ (101 MHz; CDCl_3_) 159.5, 155.9, 145.1, 144.2 (2C), 138.0, 135.9, 135.2, 134.7, 129.9, 129.6 (2C), 127.9 (2C), 121.8, 96.6, 91.6, 61.2, 58.7, 55.2, 47.9, 34.2, 30.1 (3C), 26.5, 21.0 and 20.5; LCMS *m/z* 488 [M + H]^+^; HPLC purity: 99.4%; t_r’_ = 16.99 min.

*N-{5-[(1-tert-butyl-1H-tetrazol-5-yl)(3,4-dichlorophenyl)methylamino]pentan-2-yl}-6-methoxyquinolin-8-amine***6d**, as a thick yellow oil (100.5 mg, 17%); R_f_ (DCM: acetone, 95: 0.5%) 0.29; IR ν_max_ (DCM)/cm^−1^ 1609 (Ar C = C), 1383 (N = N), 1330 (C = N), 1264 (C-N), 1245 (C-O ester); δ_H_ (400 MHz; CDCl_3_) 8.53 (1H, m, H2), 7.94 (1H, dd, *J* 8.3 and 1.6 Hz, H4), 7.42 (1H, d, *J* 2.1 Hz, H15), 7.42 (1H, d, *J* 2.1 Hz, H16), 7.38 (1H, s, H17), 7.32 (1H, dd, *J* 8.2 and 4.2 Hz, H3), 6.35 (1H, d, *J* 1.9 Hz, H7), 6.28 (1H, d, *J* 2.2 Hz, H5), 6.03 (1H, m, NH), 5.22 (1H, d, *J* 9.6 Hz, H14), 3.91 (3H, d, *J* 1.4 Hz, OCH_3_), 3.62 (1H, m, H9), 2.56 (2H, m, H13), 1.69 (4H, m, H11 and H12), 1.63 (9H, d, *J* 12.1 Hz, 3 × H18), 1.25 (3H, d, *J* 6.4 Hz, H10); δ_C_ (101 MHz; CDCl_3_) 159.5, 155.0, 145.0, 144.3, 139.1, 135.4, 134.7, 132.6, 130.8, 130.1, 130.0, 129.9, 127.3, 121.8, 96.6, 91.6, 61.3, 57.8, 55.2, 47.9, 34.0, 33.7, 30.0 (3C), 26.5 and 20.8; LCMS *m/z* 542.0 [M + H]^+^; HPLC purity: 99.5%; t_r’_ = 18.28 min.

*N-{5-[(1-tert-butyl-1H-tetrazol-5-yl)(4-chlorophenyl)methylamino]pentan-2-yl}-6-methoxyquinolin-8-amine***6e**, as a thick yellow oil (228.5 mg, 41%); R_f_ (DCM: acetone, 95: 0.5%) 0.52; IR ν_max_ (DCM)/cm^−1^ 1630 (Ar C = C), 1380 (N = N), 1335 (C = N), 1278 (C-N), 1246 (C-O ester);δ_H_ (400 MHz; CDCl_3_) 8.52 (1H, dd, *J* 4.2 and 1.6 Hz, H2), 7.93 (1H, dd, *J* 8.4 and 1.6 Hz, H4), 7.26–7.42 (5H, m, ArH and H3), 7.35 (1H, d, *J* 2.4 Hz, H7), 6.28 (1H, d, *J* 2.4 Hz, H5), 6.03 (1H, m, NH), 5.24 (1H, d, *J* 12.0 Hz, H14), 3.90 (3H, d, *J* 2.2 Hz, OCH_3_), 3.63 (1H, m, H9), 2.55 (2H, m, H13), 1.68 (4H, m, H11 and H12), 1.60 (9H, d, *J* 6.1 Hz, 3 × H17), 1.30 (3H, d, *J* 6.4 Hz, H10); δ_C_ (101 MHz; CDCl_3_) 159.5, 155.4, 145.0, 144.2 (2C), 137.4, 135.4, 134.7, 134.2, 129.9, 129.4 (2C), 129.1 (2C), 121.8, 96.6, 91.6, 61.3, 58.2, 55.2, 47.8, 34.1, 30.0 (3C), 26.5 and 20.6; LCMS *m/z* 508 [M + H]^+^; HPLC purity: 99.7%; t_r’_ = 17.25 min.

### 3.3. In Vitro Biological Evaluations

Antiplasmodial activity on the K1 strain, antitrypanosomal activity on *T. b. brucei rhodesiense* STIB900 and cytotoxicity on the L6 mammalian cell-line were all carried out at the Swiss Tropical and Public Health Institute (STPH), Switzerland as follows:

#### 3.3.1. Activity Against K1 Strain of *P. falciparum* 

In vitro activity against erythrocytic stages of *P. falciparum* was determined using a ^3^H-hypoxanthine incorporation assay, using the chloroquine and pyrimethamine-resistant K1 strain that originates from Thailand and the standard drug chloroquine (Sigma C6628) [39,40,41]. Compounds were dissolved in DMSO at 10 mg/mL and added to parasite cultures incubated in RPMI 1640 medium without hypoxanthine, supplemented with HEPES (5.94 g/L), NaHCO_3_ (2.1 g/L), neomycin (100 U/mL), Albumax^R^ (5 g/L) and washed human red cells A^+^ at 2.5% hematocrit (0.3% parasitemia). Serial drug dilutions of eleven three-fold dilution steps covering a range from 100 to 0.002 μg/mL were prepared. The 96-well plates were incubated in a humidified atmosphere at 37 °C; 4% CO_2_, 3% O_2_, 93% N_2_. After 48 h, 50 μL of ^3^H-hypoxanthine (= 0.5 μCi) was added to each well of the plate. The test compounds were tested in triplicate ad parasite growth compared to control or blank well. The plates were incubated for a further 24 h under the same conditions. The plates were then harvested with a Betaplate™ cell harvester (Wallac, Zurich, Switzerland), and the red blood cells transferred onto a glass fiber filter then washed with distilled water. The dried filters were inserted into a plastic foil with 10 mL of scintillation fluid and counted in a Betaplate™ liquid scintillation counter (Wallac, Zurich, Switzerland). IC_50_ values were calculated from sigmoidal inhibition curves by linear regression using Microsoft Excel.

#### 3.3.2. Activity against *Trypanosoma brucei rhodesiense* STIB900 

This stock was isolated in 1982 from a human patient in Tanzania and, after several mouse passages, was cloned and adapted to axenic culture conditions [41,42,43]. Minimum essential medium (50 µL) supplemented with 25 mM HEPES, 1 g/L additional glucose, 1% MEM non-essential amino acids (100×), 0.2 mM 2-mercaptoethanol, 1 mM Na-pyruvate and 15% heat-inactivated horse serum was added to each well of a 96-well microtiter plate. Serial drug dilutions of eleven three-fold dilution steps covering a range from 100 to 0.002 μg/mL were prepared. Then, 4 × 10^3^ bloodstream forms of *T. b. rhodesiense* STIB 900 in 50 µL was added to each well and the plate incubated at 37 °C under a 5% CO_2_ atmosphere for 70 h. 10 µL alamarBlue (resazurin, 12.5 mg in 100 mL double-distilled water) was then added to each well and incubation continued for a further 2–4 h. Then the plates were read with a Spectramax Gemini XS microplate fluorometer (Molecular Devices Cooperation, Sunnyvale, CA, USA) using an excitation wavelength of 536 nm and an emission wavelength of 588 nm. The test compounds were tested in triplicate, and their IC_50_ values were calculated by linear regression from the sigmoidal dose inhibition curves using Softmax Pro software (Molecular Devices Cooperation, Sunnyvale, CA, USA).

#### 3.3.3. In Vitro Cytotoxicity with L-6 Cells

Assays were performed in triplicate in 96-well microtiter plates, each well containing 100 μL of RPMI 1640 medium supplemented with 1% L-glutamine (200 mM) and 10% fetal bovine serum, and 4000 L-6 cells (a primary cell line derived from rat skeletal myoblasts) [41,44,45]. Serial drug dilutions of eleven three-fold dilution steps covering a range from 100 to 0.002 μg/mL were prepared. After 70 h of incubation, the plates were inspected under an inverted microscope to assure the growth of the controls and sterile conditions. 10 μL of alamarBlue was then added to each well, and the plates incubated for another 2 h. Then the plates were read with a Spectramax Gemini XS microplate fluorometer (Molecular Devices Cooperation, Sunnyvale, CA, USA) using an excitation wavelength of 536 nm and an emission wavelength of 588 nm. The IC_50_ values were calculated by linear regression from the sigmoidal dose inhibition curves using Softmax Pro software (Molecular Devices Cooperation, Sunnyvale, CA, USA).

#### 3.3.4. Aqueous Kinetic Solubility 

A 10 mM stock of the compound was diluted in DMSO to give standard solutions at 220 µM, 100 µM and 10 µM in a 96 well plate as described previously [19]. The 10 mM stock was then spiked 1:50 in phosphate-buffered saline pH 7.0 to give a final DMSO concentration of 2%. Both the standard and sample solutions were plated in triplicate. After shaking for 2 h at 25 °C, the solutions were filtered and analyzed on an Agilent 1200 series HPLC with DAD detection. The solubility of the test compounds was determined by comparing the peak area of the sample solutions to that of the standard solutions.

## 4. Conclusions

In this study, we presented the synthesis of 4-amino-7chloroquinoline tethered β-lactams and primaquine-based tetrazoles, the latter obtained as unresolved 1:1 diastereomeric mixtures. The configuration of the β-lactams was assigned as *cis* based on the NMR analysis. The in vitro antiplasmodium activity of all the β-lactams was superior to those of the primaquine-based tetrazoles and primaquine reference drug, with compounds **4d**–**e and 4g** being two to three-fold more active than primaquine while **4d** was also equipotent to chloroquine. The most active compound in the tetrazole series, **6d**, was 2- and 6-fold less active than primaquine and chloroquine, respectively. The antitrypanosomal activity of all the synthesized compounds was moderate, with compounds **4d**, **4e**–**g** and **6a**–**d** displaying encouraging antitrypanosomal activities less than 20 µM. Furthermore, three β-lactams and three tetrazoles were assessed for their aqueous kinetic solubility and generally, the β-lactams compounds exhibited superior solubility (>200 µM) compared to those of tetrazoles (<20 µM).

## Figures and Tables

**Table 1:** In vitro antiplasmodium and antitrypanosomal activity, and cytotoxicity of the synthesized target compounds.

Compound	*n*	R	R^1^	*P. falciparum* (µM) *	*T. b. brucie* (µM) *	Cytotoxicity (µM) *	SI ^#^
				K1	*T. b. rhod*	L6 Cell Lines	
**4a**	**1**	**H**	**H**	0.47	53.16	98.47	211
**4b**	**1**	OCH_3_	H	0.62	52.93	20.37	33
**4c**	**1**	Me	H	0.58	52.54	92.70	160
**4d**	**1**	Cl	Cl	0.20	18.99	8.54	43
**4e**	**1**	Cl	H	0.30	7.01	11.52	38
**4f**	**2**	H	H	0.57	16.09	14.40	25
**4 g**	**3**	H	H	0.31	20.00	2.87	9
**6a**	-	H	H	2.47	18.10	10.77	4
**6b**	-	OCH_3_	H	1.98	14.16	75.45	38
**6c**	--	CH_3_	H	1.57	18.54	12.94	8
**6d**	-	Cl	Cl	1.31	18.30	87.93	67
**6e**	-	Cl	H	1.80	21.45	31.30	17
**Chloroquine**	-	-	-	0.22	-	-	-
**Primaquine**	-	-	-	0.64	-	-	-
**Melarsoprol**	-	-		-	0.0075	-	-
**Podophyllotoxin**	-	-		-	-	0.0193	-

* All IC_50_; ^#^ Selectivity Indices (SI) [(IC_50_ L6 Cell Line)/(IC_50_ K1 strain)].

**Table 2:** Aqueous kinetic solubility of selected compounds.

Compound	Kinetic Solubility at pH 7.0 (µM)
**4a**	>200
**4e**	>200
**4g**	>200
**6c**	<5
**6d**	<5
**6e**	<15

## Data Availability

Sample of compounds reported in this study are available on request from the corresponding author.

## Supplementary Materials

The following are available online. Table S1: Yields and HPLC purity of the synthesized compounds; preparative and analytical HPLC methods, HPLC chromatograms for 1:1 diastereomeric mixtures (Figures S1–S5), ^1^H and ^13^C NMR spectra of the synthesized target compounds.

## References

[1] Filardy A.A., Guimarães-Pinto K., Nunes M.P., Zukeram K., Fliess L., Oliveira-Nascimento D., Conde L., Morrot A. (2018). Human kinetoplastic protozoan infections: Where are we going next?. Front. Immunol..

[2] Njoroge A., Njuguna N.M., Mutai P., Ongaror D.S., Smith P.W., Chibale K. (2014). Recent approaches to chemical discovery and development against malaria and the neglected tropical diseases human African trypanosomiasis and schistosomiasis. Chem. Rev..

[3] Rao S.P.S., Barrett M.P., Dranoff G., Faraday C.J., Gimpelewicz C.R., Hailu A., Jones C.L., Kelly J.M., Lazdins-Helds J.K., Mäser P. (2019). Drug discovery for kenetoplastic diseases: Future directions. ACS Infect. Dis..

[4] Xie S.C., Dick L.R., Gould A., Brand S., Tilley L. (2019). The proteasome as a target for protozoan parasites. Expert Opin. Ther. Targets..

[5] Kennedy P.G.E. (2019). Update on human African trypanosomiasis (Sleeping sickness). J. Neurol..

[6] WHO Report, World Malaria Report 2019. https://www.who.int/publications-detail/world-malaria-report-2019.

[7] WHO Report, Fourth WHO Report on Neglected Tropical Diseases 2017. https://www.who.int/neglected_diseases/resources/9789241565448/en/.

[8] Akazue P.I., Ebiloma G.U., Ajibola O., Isaac C., Onyekwelu K., Ezeh C.O., Eze A.A. (2019). Sustainable elimination (Zero cases) of sleeping sickness: How far are we from achieving this goal?. Pathogen.

[9] Capewell P., Atkins K., Weir W., Jamonneau V., Camara M., Clucas C., Swar N.-R.K., Ngoyi D.M., Routureau B., Garside P. (2019). Resolving the apparent transmission paradox of African sleeping sickness. PLoS Biol..

[10] Jacobs R.T., Nare B., Phillips M.A. (2011). State of the art African trypanosome drug discovery. Curr. Top Med. Chem..

[11] Fairlamb A.H., Horn D. (2018). Melarsoprol resistance in African trypanosomiasis. Trends Parasitol..

[12] Lee S.M., Kim M.S., Hayat F., Shin D. (2019). Recent advances in the discovery of novel antiprotozoal agents. Molecules.

[13] Van Voorhis W.C., Adams J.H., Adelfio R., Ahuong V., Akabas M.H., Alano P., Alday A., Resto Y.A., Alsibaee A., Alzualde A. (2016). Open source drug discovery with malaria box compound collection for neglected diseases and beyond. PLoS Pathog..

[14] Berniger M., Scmidt I., Ponte-Sucre A., Holzgrabe U. (2017). Novel lead compounds in pre-clinical development against African sleeping sickness. MedChemComm.

[15] Ashley E.A., Dhorda M., Fairhurst R.M., Amaratunga C., Lim P., Suon S., Sreng S., Anderson J.M., Mao S., Sam B. (2014). Spread of artemisinin resistance in *Plasmodium falciparum* malaria. N. Engl. J. Med..

[16] Mbengue A., Bhattacharjee S., Pandharkar T., Liu H., Estiu G., Stahelin R.V., Rizk S.S., Njimoh D.L., Ryan Y., Chotivanich K. (2015). Amolecular mechanism of artemisinin resistance in *Plasmodium falciparum* malaria. Nature.

[17] Fairhurst R.M., Dondorp A.M. (2016). Atermisinin-resistant *Plasmodium falciparum* malaria. Microbiol. Spectr..

[18] Dondorp A.M., Yeung S., White L., Nguon C., Day N.P., Socheat D., von Seidlein L. (2010). Artemisinin-resistance: Current status and scenarios for containment. Nat. Rev. Microbiol..

[19] Tukulula M., Njoroge M., Abay E.T., Mugumbate G.C., Wiesner L., Taylor D., Gibhard L., Norman J., Swart K.J., Gut J. (2013). Synthesis and in vitro and in vivo pharmacological evaluation of new 4-aminiquinoline-based compounds. ACS Med. Chem. Lett..

[20] Abay E.T., van der Westhuizen J.H., Swart K.J., Gibhard L., Tukulula M., Chibale K., Wiesner L. (2014). The development and validation of an LC-MS/MS method for the determination of a new antimalarial compound (TK900D) in human whole blood and its application to pharmacokinetic studies in mice. Malar. J..

[21] Nivsarkar M., Thavaselvam D., Prasanna S., Sharma M., Kaushik M.P. (2005). Design, synthesis and biological evaluation of novel bicyclic β-lactams as potential antimalarials. Bioor. Med. Chem. Lett..

[22] D’hooghe M., Dekeukeleire S., Mollet K., Lategan C., Smith P.J., Chibale K., De Kimpe N. (2009). Synthesis of novel 2-alkoxy-3-amino-3-arylpropan-1-ols and 5-alkoxy-4-aryl-1,3-oxazinanes with antimalarial activity. J. Med. Chem..

[23] Singh P., Sachedeva S., Raj R., Kumar V., Mahajan M.P., Nasser S., Vivas L., Gut J., Rosenthal P.J., Feng T.-S. (2011). Antiplasmodial and cytotoxicity evaluation of 3-functionalized 2-azetedinone derivatives. Bioorg. Med. Chem. Lett..

[24] Raj R., Biot C., Carrére-Kremer S., Kremer L., Guérardel Y., Gut J., Rosenthal P.J., Kumar V. (2014). 4-Aminoquinoline-β-lactam conjugates: Synthesis, antimalarial, and antitubercular evaluation. Chem. Biol. Drug Des..

[25] Singh P., Singh P., Kumar M., Gut J., Rosenthal P.J., Kumar K., Kumar V., Mahajan M.P., Bissetty K. (2012). Synthesis, docking and in vitro antimalarial evaluation of bifunctional hybrids derived from β-lactams and 7-chloroquinoline using click chemistry. Bioorg. Med. Chem. Lett..

[26] Jarrahpour A., Ebrahimi E., Sinou V., Latour C., Brunel J.M. (2014). Diastereselective synthesis of potent antimalarial *cis*-β-lactam agents through a [2 + 2] cycloaddition of chiral imines with a chiral ketene. Eur. J. Med. Chem..

[27] Jarrahpour A., Aye M., Sinou V., Latour C., Brunel J.M. (2015). Synthesis of some new monocyclic β-lactams as antimalarial agents. J. Iran Chem..

[28] Staudinger H. (1907). Zur Kenntniss der keten. Diphenylketen. Liebigs Ann. Chem..

[29] Musonda C.C., Gut J., Rosenthal P.J., Yardley V., Carvalho de Souza R.C., Chibale K. (2006). Application of multicomponent reactions to antimalarial drug discovery. Part 2: New antiplasmodial and antitrypanosomal 4-aminoquinoline δ- and ϒ-lactams via a ‘catch and release’ protocol. Bioorg. Med. Chem..

[30] McCabe R.E. (1988). Primaquine is lethal for intracellular but not for extracellular trypanosome cruzi. J. Parasitol..

[31] Carvalho L., Martínez-Garcia M., Pérez-Victoria L., Manzano J.I., Yardley V., Gamarro F., Pérez-Victoria M. (2015). The oral antimalarial drug tafenoquine shows activity against trypanosome brucei. Antimicrob. Agents Chemother..

[32] Yardley V., Gamarro F., Croft S.L. (2010). Antileishmanial and antitrypanosomal activities of the 8-aminoquinoline tafenoquine. Antimicrob. Agents Chemother..

[33] De D., Byers L.D., Krogstad D.J. (1997). Antimalarial: Synthesis of 4-aminoqquinolines that circumvent resistance in malarial parasite. J. Heterocycl. Chem..

[34] Jiao L., Liang Y., Xu J. (2006). Origin of the relative stereoselectivity of the β-lactam formation in the Staudinger reaction. J. Am. Chem. Soc..

[35] Xu J. (2009). Stereoselectivity in the synthesis of 2-azetidinones from ketenes and imines via the Staudinger reaction. ARKIVOC.

[36] Tukulula M., Little S., Gut J., Rosenthal P.J., Wan B., Franzblau S.G., Chibale K. (2012). The design, synthesis, in silico ADME profiling, antiplasmodial and antimycobacterial evaluation of the new arylamino quinoline derivatives. Eur. J. Med. Chem..

[37] Tukulula M., Njoroge M., Mugumbate G.C., Gut J., Rosenthal P.J., Barteau S., Streckfuss J., Heidi O., Kameni-Tcheudji J., Chibale K. (2013). Tetrazole-based deoxyamodiaquines: Synthesis, ADME/PK profiling and pharmacological evaluation as potential antimalarial agents. Bioorg. Med. Chem..

[38] Tukulula M., Sharma R.-J., Meurillon M., Mahajan A., Naran K., Warner D., Huang J., Mekonnen B., Chibale K. (2013). Synthesis and antiplasmodial and antimycobacterial evaluation of new nitroimidazoleand nitroimizaooxazine derivatives. ACS Med. Chem..

[39] Desjardins R.E., Canfield C.J., Haynes J.D., Chulay J.D. (1979). Quantitative assessment of antimalarial activity in vitro by a semiautomated microdilution technique. Antimicrob. Agents Chemther..

[40] Thaithong S., Beale G.H., Chutmongkonkul M. (1983). Susceptibility of *Plasmodium falciparum* to five drugs and in vitro study of isolates mainly from Thailand. Trans. R. Soc. Trop. Med. Hyg..

[41] Hubber W., Koella J.C. (1993). A comparison of three methods of estimating EC50 in studies of drug resistance of malaria parasites. Acta Trop..

[42] Balts T., Baltz D., Giroud C., Crockett J. (1985). Cultivation in a semi-defined medium if animal infective forms of *Trypanosoma brucei*, *T. equiperdum*, *T. evansi*, *T. rhodesiense* and *T. gambiense*. EMBO J..

[43] Räz B., Iten M., Grether-Bühler Y., Kaminsky R., Brun R. (1997). The alma blue assay to determine drug sensitivity of African trypanosomes (*T. b. rhodesiense* and *T. b gambiense*) in vitro. Acta Trop..

[44] Page B., Page M., Noel C. (1993). A new fluorometric assay for cytotoxicity measurement in vitro. Int. J. Oncol..

[45] Ahmed S.A., Gogal R.M., Walsh J.E. (1994). A new rapid and simple non-radioactive assay to monitor and determine the proliferation of lymphocytes: An alternative to [3H] thymidine incorporation assay. J. Immunol. Methods.

