# Supplementary material for: Synthesis and In Vitro Antiprotozoan Evaluation of 4-/8-Aminoquinoline-based Lactams and Tetrazoles
Source: Molecules. 2020 Dec 15;25(24):5941. doi: 10.3390/molecules25245941 (PMC7765388; doi:10.3390/molecules25245941)
Supplement: Supplementary file 1 [file molecules-25-05941-s001.pdf]

Synthesis and In Vitro Antiprotozoan Evaluation of 4-/8-Aminoquinoline-Based Lactams and Tetrazoles

Matshawandile Tukulula,[1] Stefan Louw,[2,3] Mathew Njoroge,[2] Kelly Chibale[2,4,5]*

[1]School of Chemistry and Physics, University of KwaZulu Natal, Durban, 4000, South Africa.

[2]Department of Chemistry, University of Cape Town, Rondebosch 7701, South Africa

[3]Department of Chemistry and Biochemistry, University of Namibia, Windhoek, Namibia.

[4]Institute of Infectious Disease and Molecular Medicine, University of Cape Town, Rondebosch 7701, South Africa.

[5]South African Medical Research Council Drug Discovery and Development Research Unit, Department of Chemistry University of Cape Town, Rondebosch 7701, South Africa.

*Corresponding author: Email: tukululaM@ukzn.ac.za Tel +27-31 260 8756 Fax +27-31 260 3091;

TableS1: Yields and HPLC purity of the synthesised compounds

Compound	n	R’	R	Yield/[%]	HPLC Purity/[%][a]
**4a**	**1**	H	H	30	95.6
**4b**	**1**	H	OCH_3	9	95.6
**4c**	**1**	H	Me	6	94.6
**4d**	**1**	Cl	Cl	4	99.5
**4e**	**1**	H	Cl	14	99.2
**4f***	**2**	H	H	5*	-*
**4g**	**3**	H	H	9	98
**6a**	**-**	H	H	21	99.3
**6b**	**-**	H	OCH_3	40	99.5
**6c**	**-**	H	CH_3	50	99.4
**6d**	**-**	Cl	Cl	17	99.5
**6e**	**-**	H	Cl	41	99.7

[a]HPLC Purity was determined as an area under a curve (λ = 220 and 254 nm);*This compound still contained traces of the Schiff bases even after extensive purification

Analytical and preparative HPLC experimental conditions:

HPLC mobile phase: All HPLC separations were performed using mobile phase A, an aqueous 10 mM ammonium bicarbonate solution at pH 11, mixed on-line with mobile phase B which consisted of 10 mM ammonium bicarbonate solution (pH 11) in 90% methanol (prepared from the same 100 mM ammonium bicarbonate pH 11 buffer stock solution; pH adjusted with 25 % ammonia solution).

Preparative HPLC conditions: Sample solutions for purification were prepared at *ca.* 50 mg/mL in methanol. Injection volumes ranged between 50 µL and 1 mL, depending on the sample, the mobile phase flow rate was 20 mL/min for all purifications and the column heater was set at 30°C. A linear mobile phase gradient was used, 60 % to 100 % mobile phase B over 9 min. (remain at 100 % B for 2 min.).

Analytical HPLC conditions: Solutions for analysis were prepared at *ca.* 1 mg/mL in methanol. Injection volume for all analyses was 20 µL, the mobile phase flow rate was 1.2 mL/min and the column temperature was maintained at 30 °C. A linear gradient was used, 20 % to 100 % mobile phase B over 9 min. (remain at 100 % B for 8 min.).

Instrumentation: Both analytical and preparative HPLC separations were performed on a modular Waters HPLC system (Microsep, Tygervalley, South Africa) consisting of a 2767 sample manager, 2545 quaternary gradient pump, 1500 series column heater and a 2998 photodiode array detector (PDA) with a Prep 2998 flowcell. MassLynx software (version 4.1) was used for instrument control and data acquisition, while FractionLynx software (version 4.1) was used to control the collection of HPLC fractions. A Waters Xbridge C_{18}, 4.6 x 150 mm column with 5 µm particles was used for the analytical scale HPLC analysis and a Waters Xbridge C_{18}, OBD 19 x 250 mm preparative HPLC column was used for the and purifications. Xbridge C_{18}, 5 µm, 4.6 x 20 mm and Xbridge C_{18}, 5 µm, 19 x 10 mm guard columns were connected to the inlets of the analytical and preparative HPLC columns respectively.

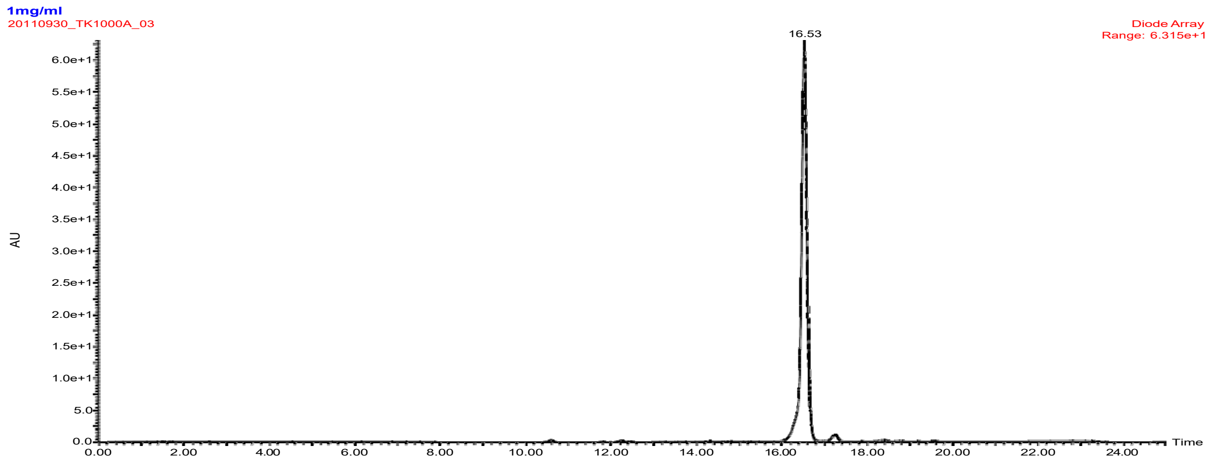

Figure S1: HPLC-PDA chromatogram of **6a**.

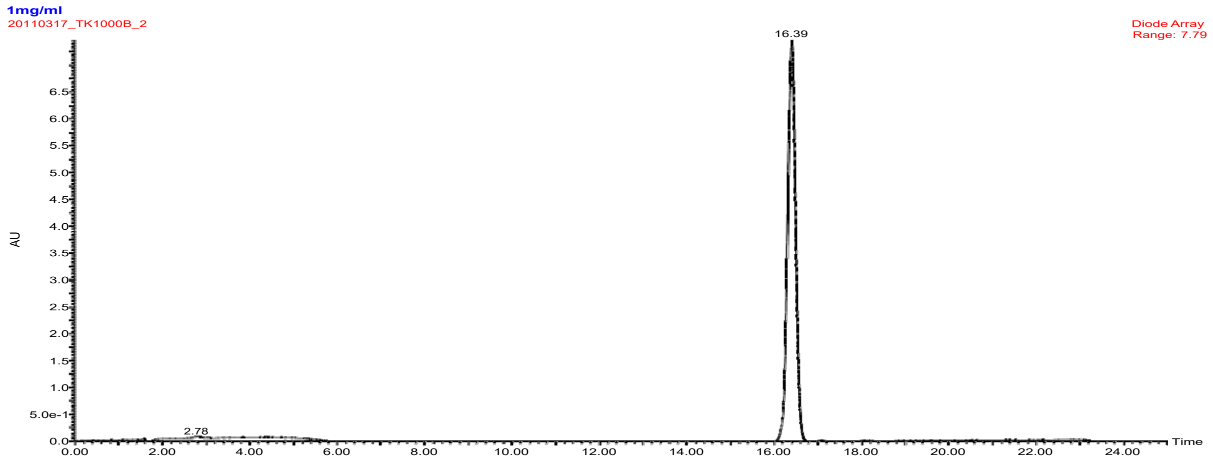

Figure S2: HPLC- PDA chromatogram of **6b**.

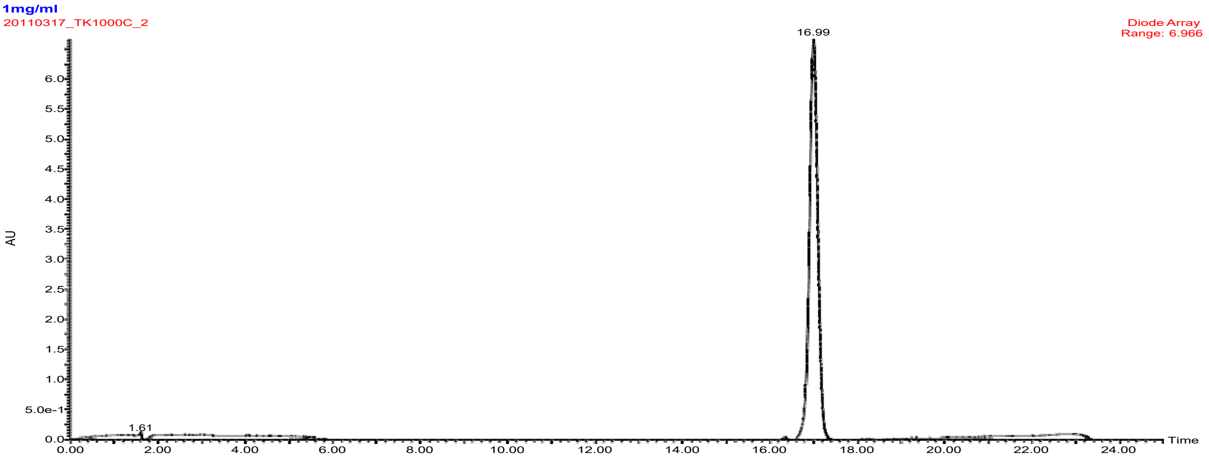

Figure S3: HPLC- PDA chromatogram of **6c**.

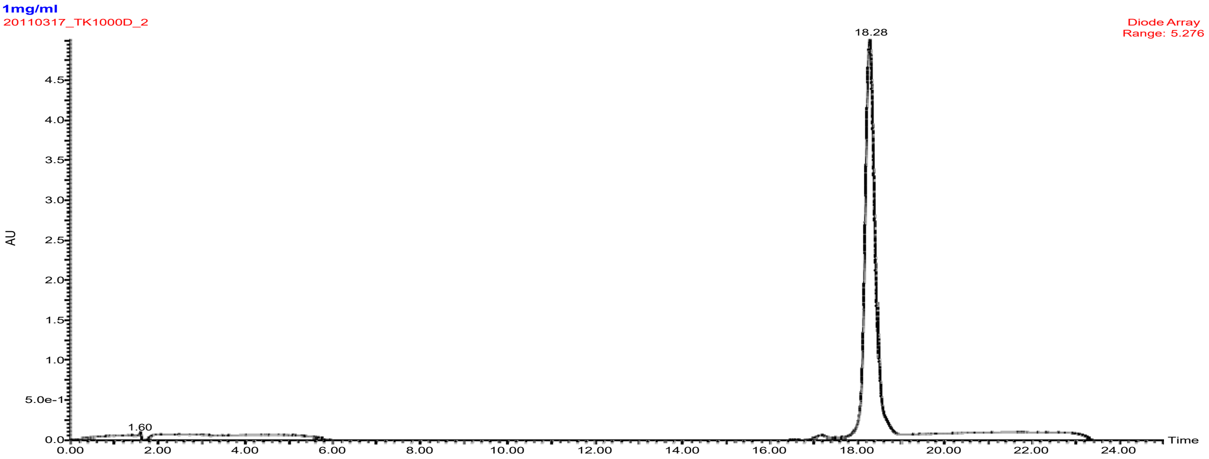

Figure S4: HPLC- PDA chromatogram of **6d**.

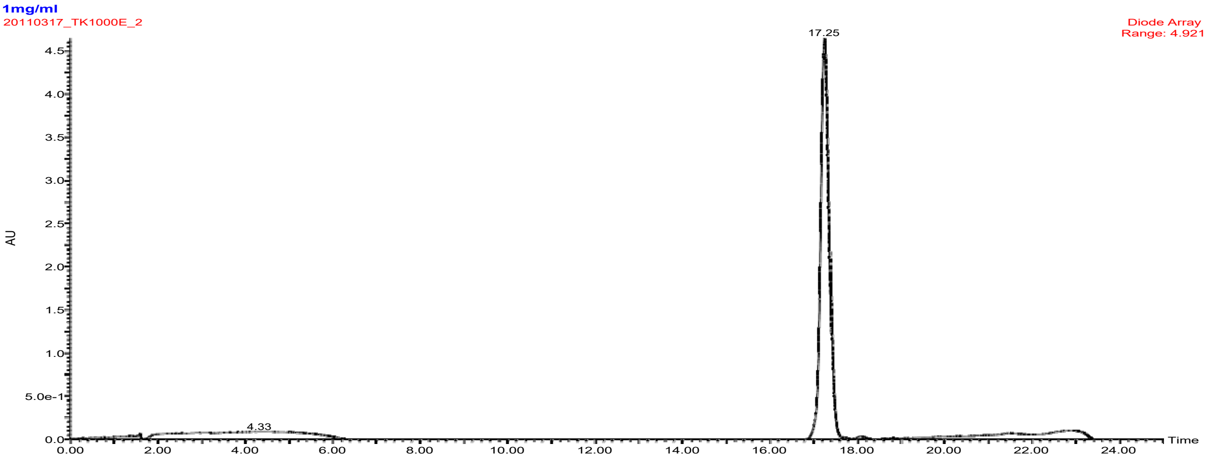

Figure S5: HPLC- PDA chromatogram of **6e**.

NMR Spectra of the target compounds:

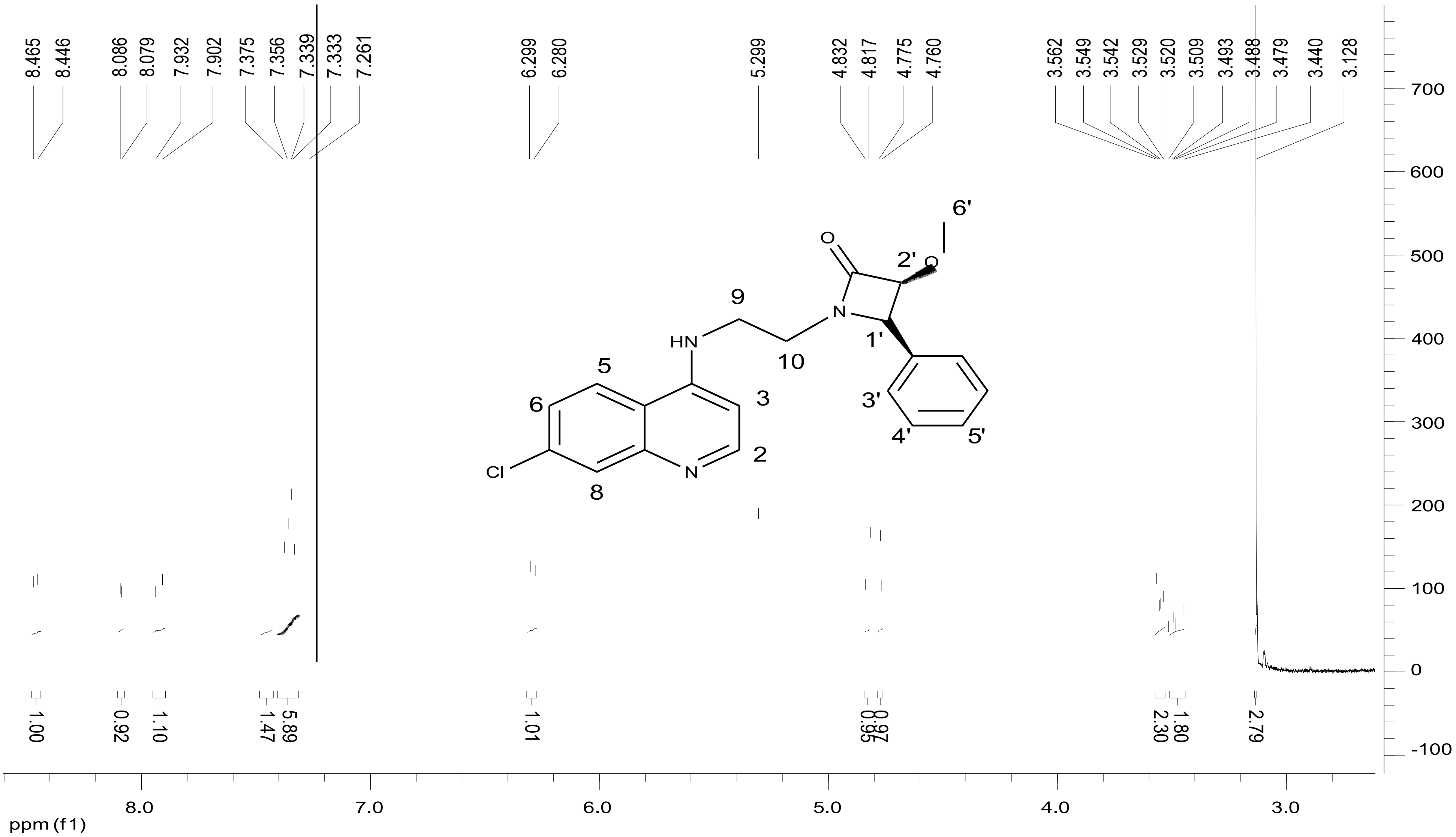

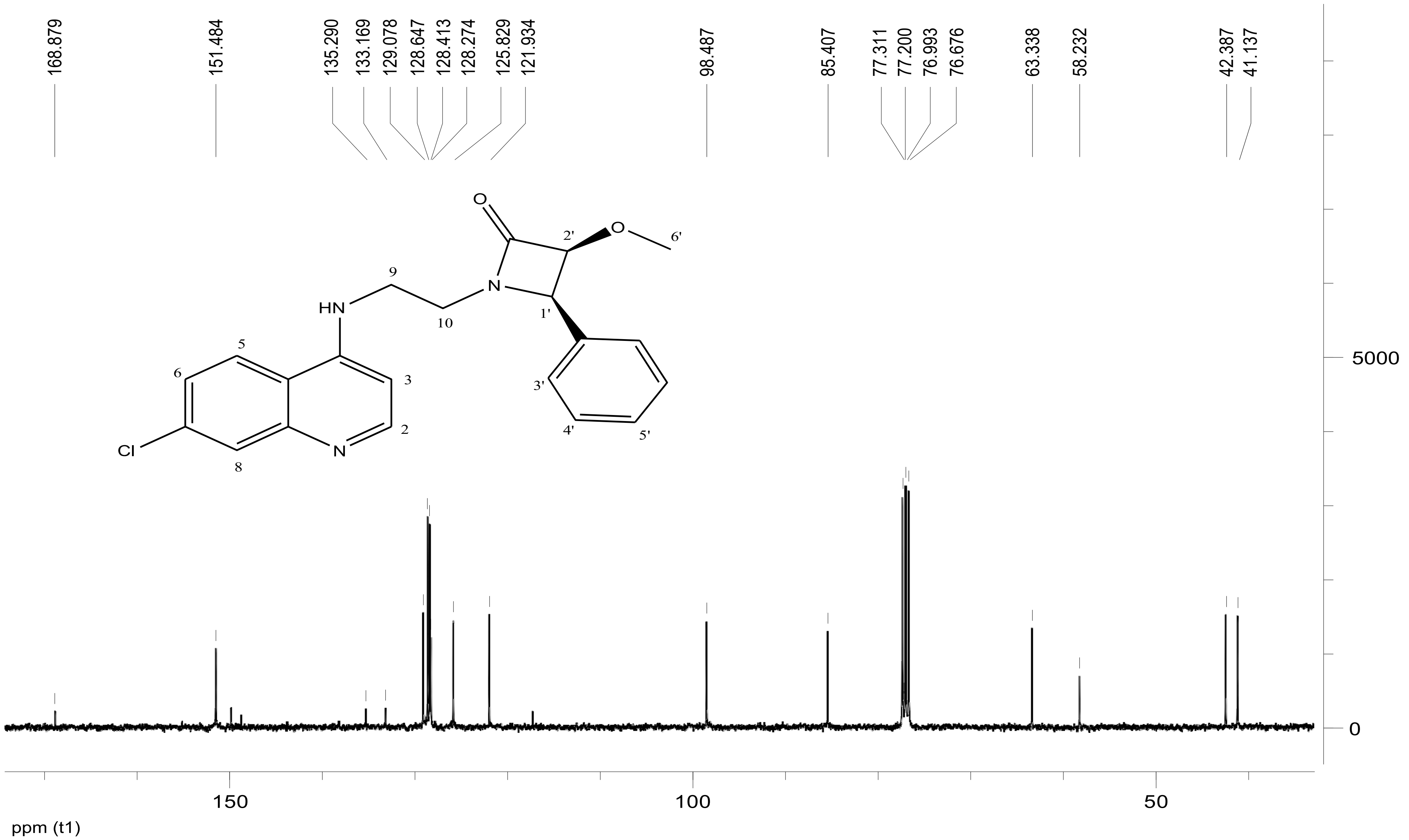

168.879
151.484
135.290
133.169
129.078
128.647
128.413
128.274
125.829
121.934
98.487
85.407
77.311
77.200
76.993
76.676
63.338
58.232
42.387
41.137
O
2'
O
6'
9
HN
N
10
1'
5
6
3
3'
2
Cl
N
4'
5'
8
5000
0
150
100
50
ppm (t1)

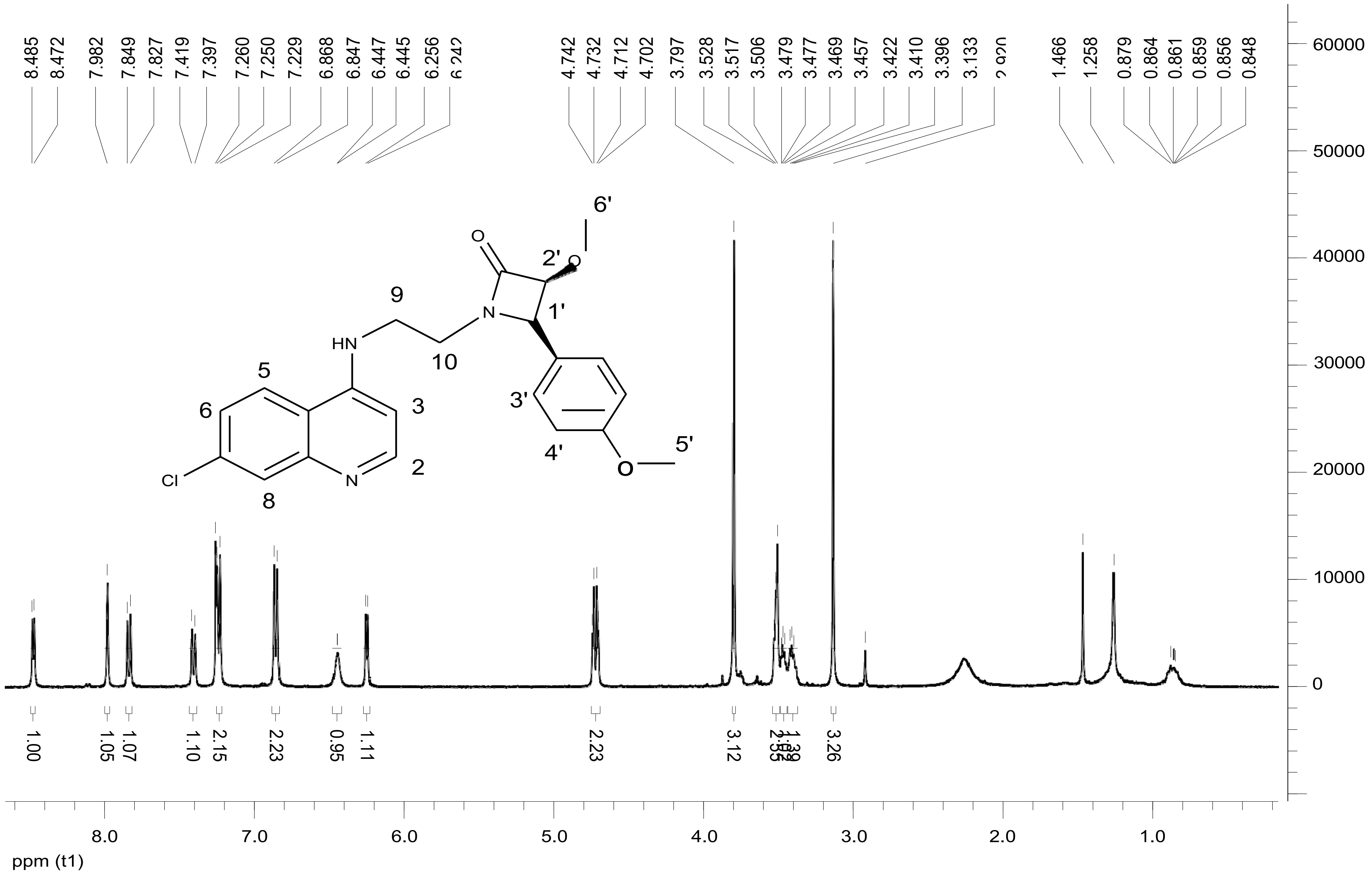

8.485
8.472
7.982
7.849
7.827
7.419
7.397
7.260
7.250
7.229
6.868
6.847
6.447
6.445
6.256
4.742
4.732
4.712
4.702
3.797
3.528
3.517
3.506
3.479
3.477
3.469
3.457
3.422
3.410
3.396
3.133
1.466
1.258
0.879
0.864
0.861
0.859
0.856
0.848
60000
50000
40000
30000
20000
10000
0
6'
O
2'
O
9
N
1'
HN
10
5
3
3'
6
5'
4'
2
O
Cl
N
8
1.00
1.05
1.07
1.10
2.15
2.23
0.95
1.11
2.23
3.12
3.26
8.0
7.0
6.0
5.0
4.0
3.0
2.0
1.0
ppm (t1)

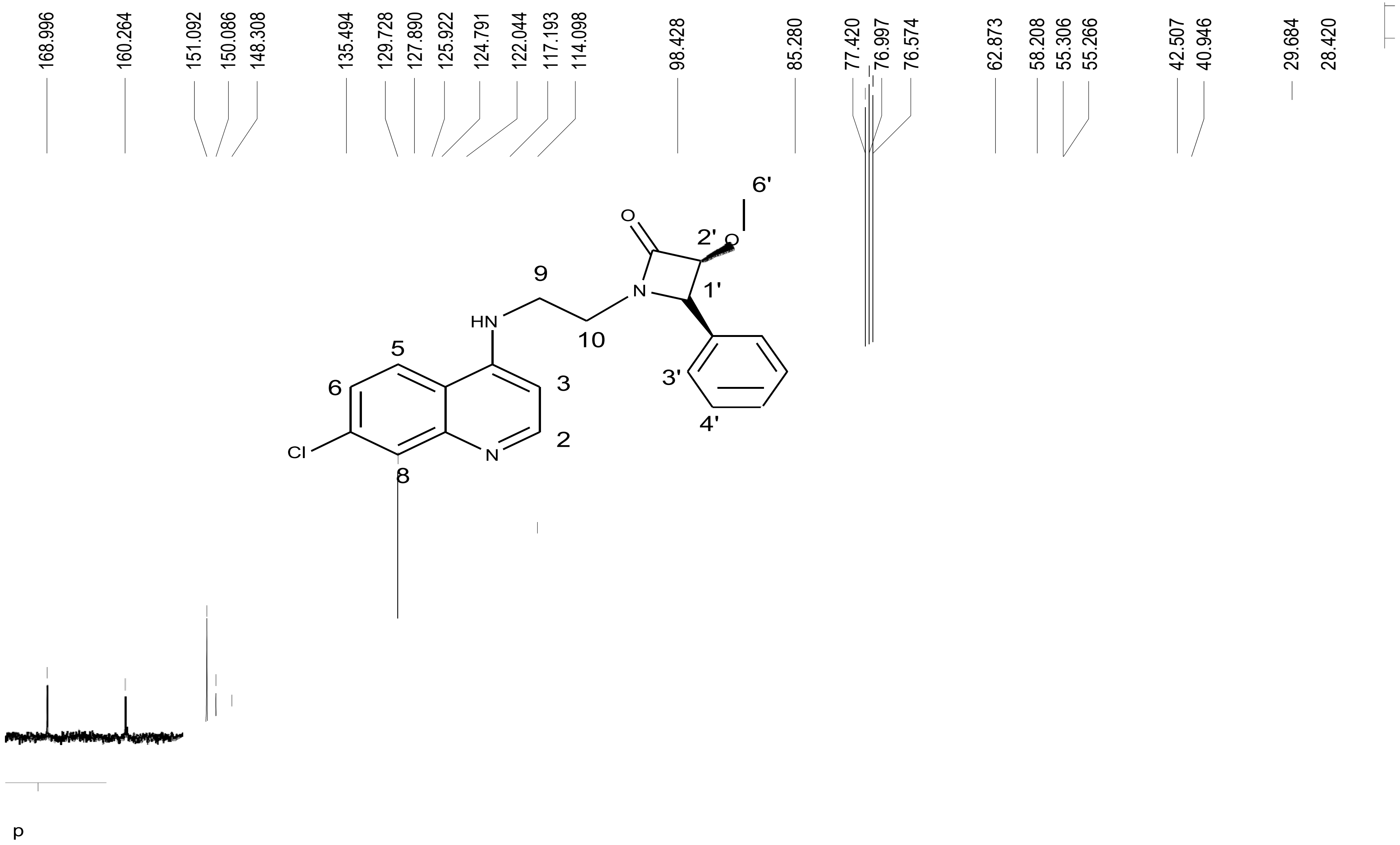
168.996
160.264
151.092
150.086
148.308
135.494
129.728
127.890
125.922
124.791
122.044
117.193
114.098
98.428
85.280
77.420
76.997
76.574
62.873
58.208
55.306
55.266
42.507
40.946
29.684
28.420
6'
O
2'
O
9
1'
N
HN
10
5
6
3
3'
4'
2
Cl
N
8
p

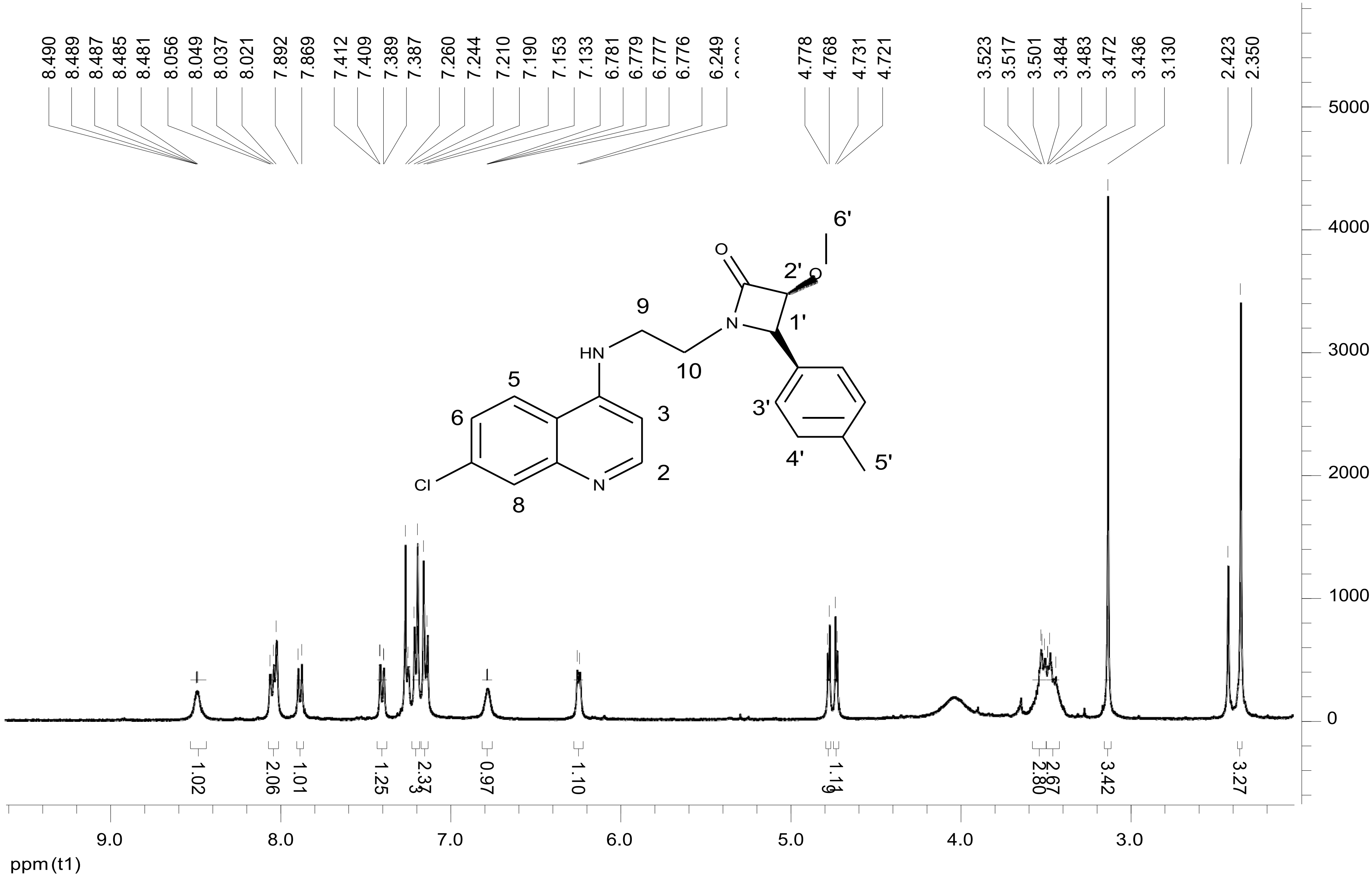

8.490
8.489
8.487
8.485
8.481
8.056
8.049
8.037
8.021
7.892
7.869
7.412
7.409
7.389
7.387
7.260
7.244
7.210
7.190
7.153
7.133
6.781
6.779
6.777
6.776
6.249
4.778
4.768
4.731
4.721
3.523
3.517
3.501
3.484
3.483
3.472
3.436
3.130
2.423
2.350
5000
4000
3000
2000
1000
0
O
6'
2'
9
N
1'
HN
10
5
3
3'
6
2
4'
5'
Cl
N
8
1.02
2.06
1.01
1.25
2.37
0.97
1.10
1.11
2.80
2.67
3.42
3.27
9.0
8.0
7.0
6.0
5.0
4.0
3.0
ppm (t1)

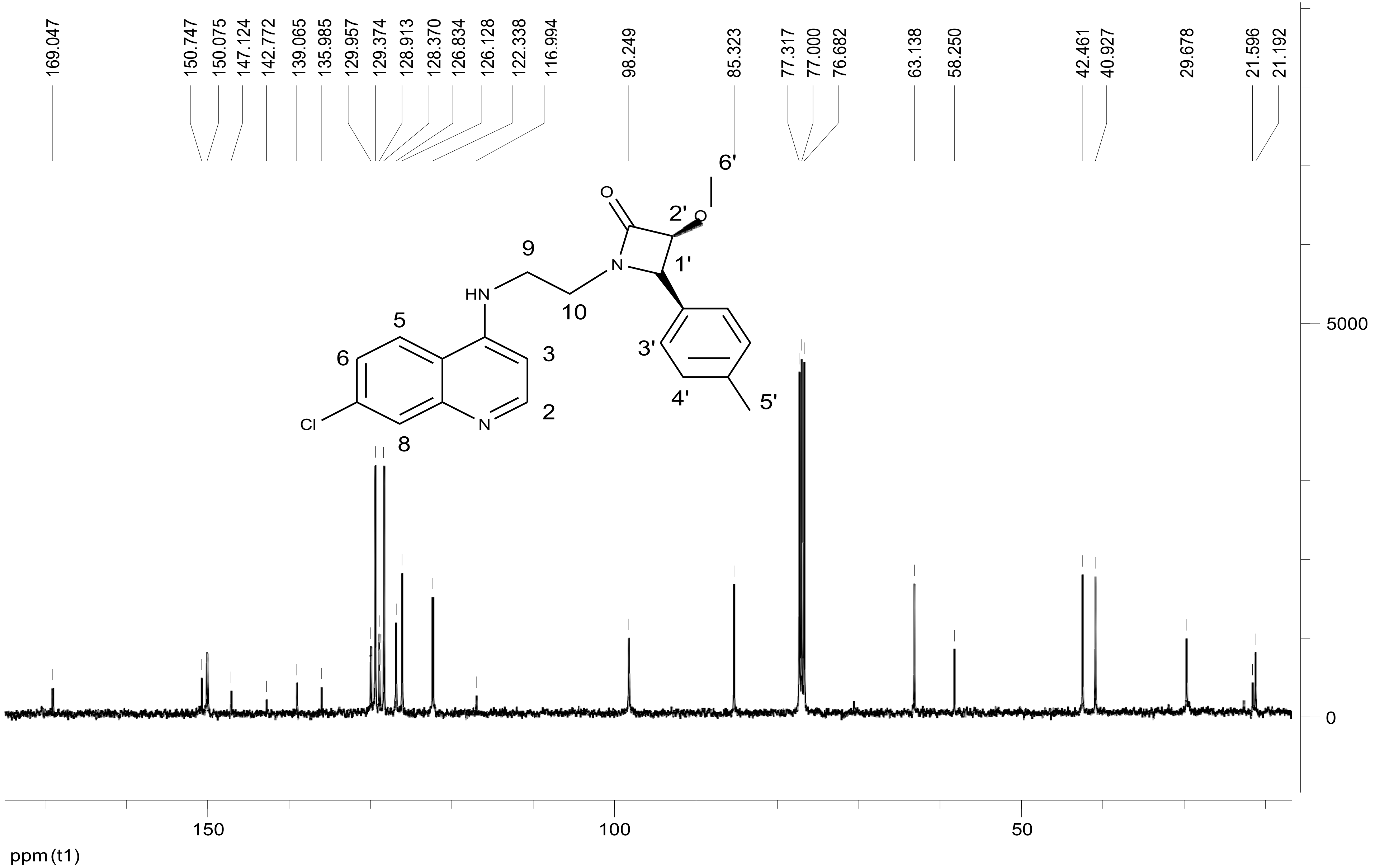
169.047
150.747
150.075
147.124
142.772
139.065
135.985
129.957
129.374
128.913
128.370
126.834
126.128
122.338
116.994
98.249
85.323
77.317
77.000
76.682
63.138
58.250
42.461
40.927
29.678
21.596
21.192
6'
O
2'
O
9
N
1'
HN
10
5
3
6
3'
2
4'
5'
Cl
N
8
5000
0
150
100
50
ppm (t1)

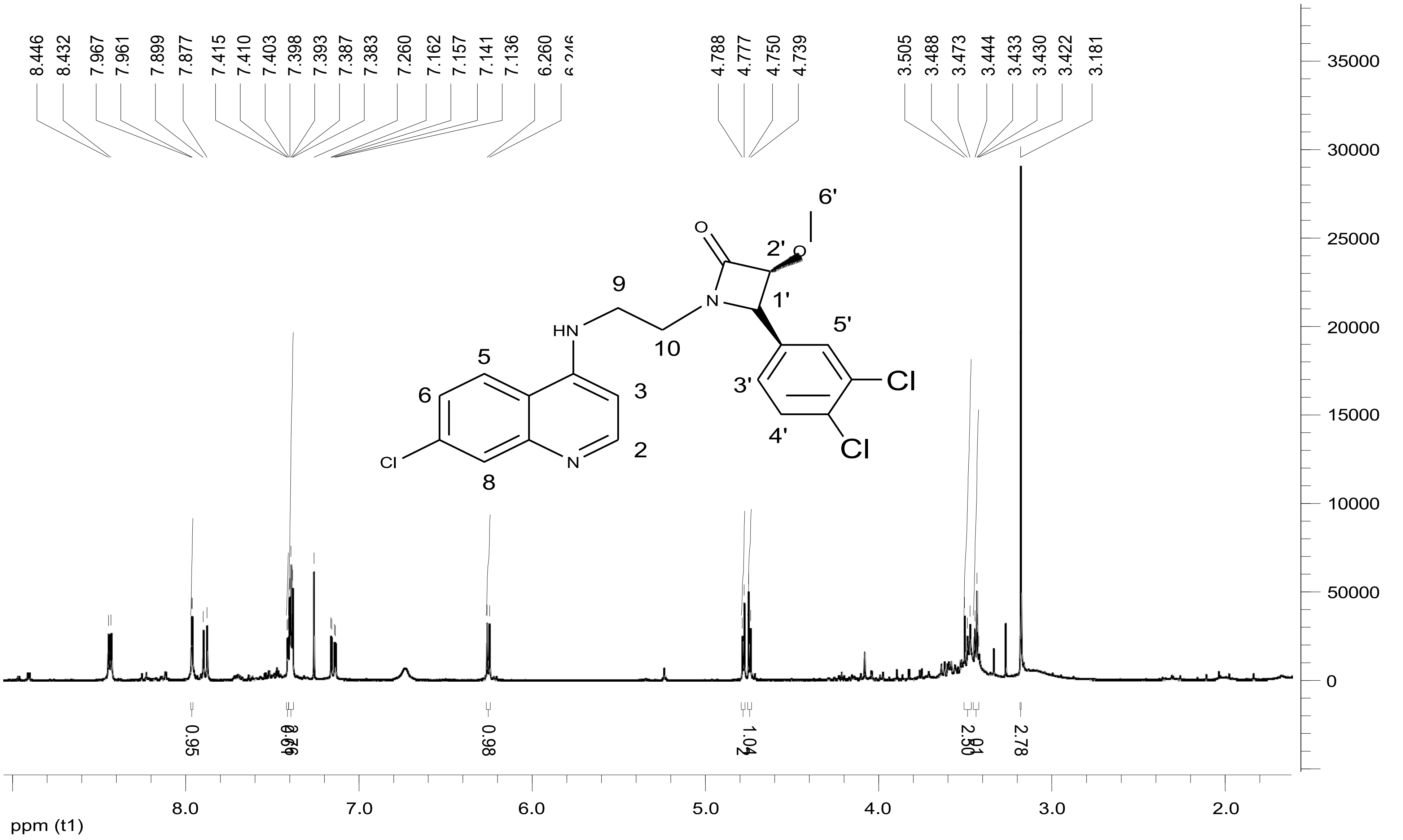
8.446
8.432
7.967
7.961
7.899
7.877
7.415
7.410
7.403
7.398
7.393
7.387
7.383
7.260
7.162
7.157
7.141
7.136
6.260
4.788
4.777
4.750
4.739
3.505
3.488
3.473
3.444
3.433
3.430
3.422
3.181
0.95
0.98
2.78
ppm (t1)
8.0
7.0
6.0
5.0
4.0
3.0
2.0
35000
30000
25000
20000
15000
10000
50000
0
O
6'
2'
9
1'
HN
N
5'
10
5
3
3'
Cl
6
4'
2
Cl
Cl
N
8

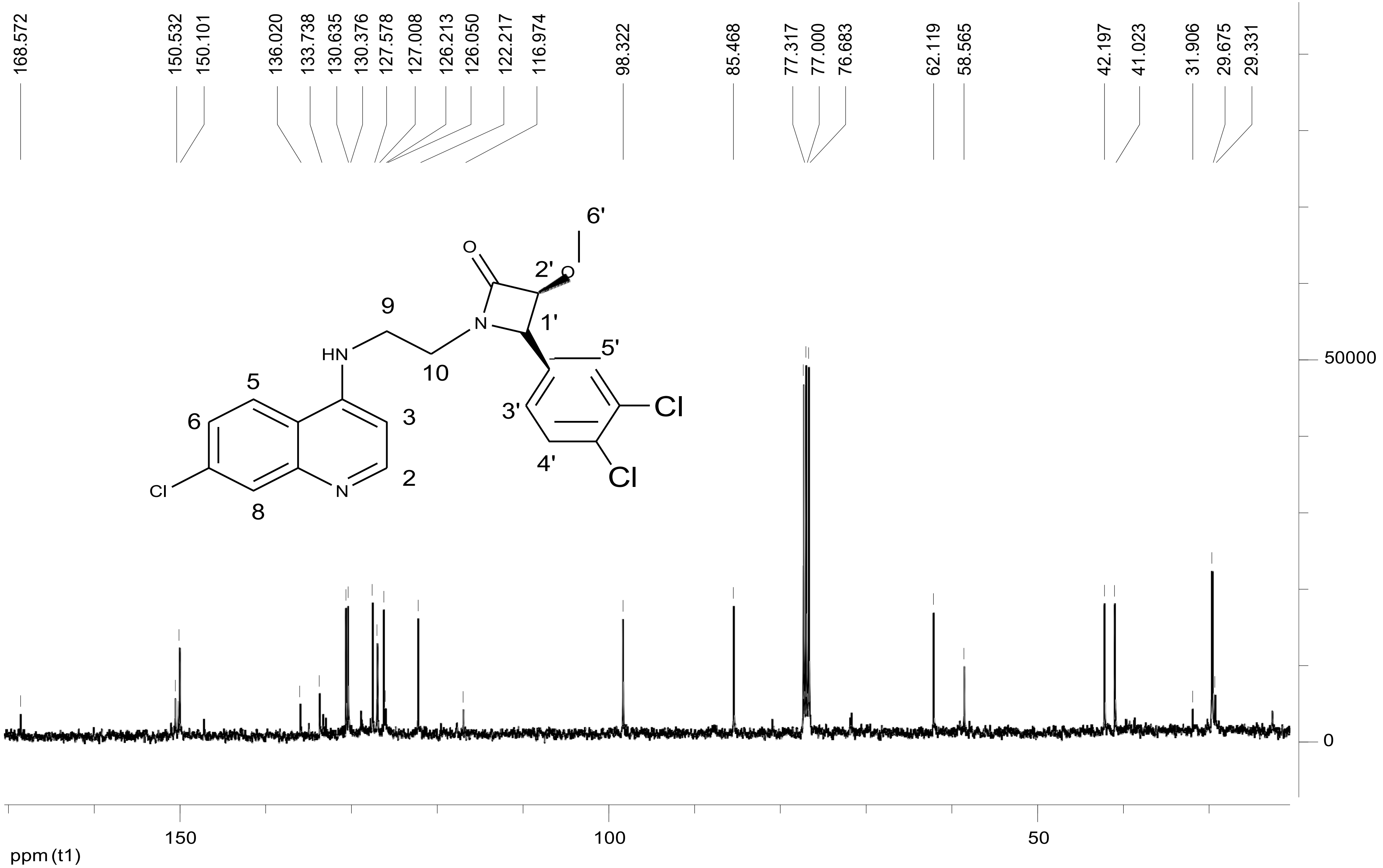

168.572
150.532
150.101
136.020
133.738
130.635
130.376
127.578
127.008
126.213
126.050
122.217
116.974
98.322
85.468
77.317
77.000
76.683
62.119
58.565
42.197
41.023
31.906
29.675
29.331
6'
O
2'
O
9
1'
N
HN
5'
10
5
3
3'
Cl
6
Cl
4'
2
Cl
N
8
50000
0
150
100
50
ppm (t1)

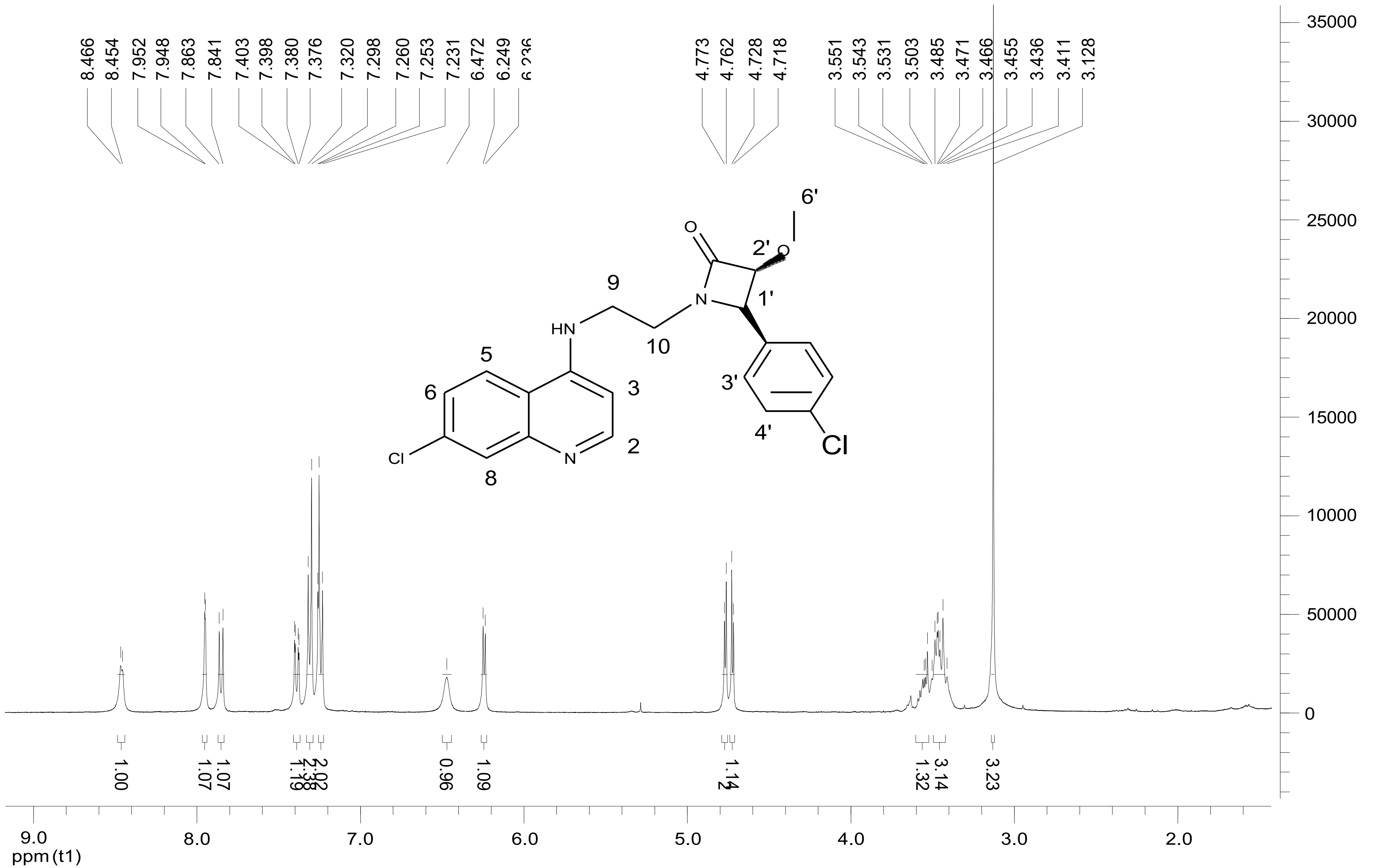
8.466
8.454
7.952
7.948
7.863
7.841
7.403
7.398
7.380
7.376
7.320
7.298
7.260
7.253
7.231
6.472
6.249
4.773
4.762
4.728
4.718
3.551
3.543
3.531
3.503
3.485
3.471
3.466
3.455
3.436
3.411
3.128
6'
O
2'
O
9
1'
N
HN
10
5
6
3
3'
Cl
N
2
4'
Cl
8
35000
30000
25000
20000
15000
10000
50000
0
1.00
1.07
1.07
1.19
2.38
2.02
0.96
1.09
1.14
1.32
3.14
3.23
9.0
8.0
7.0
6.0
5.0
4.0
3.0
2.0
ppm (t1)

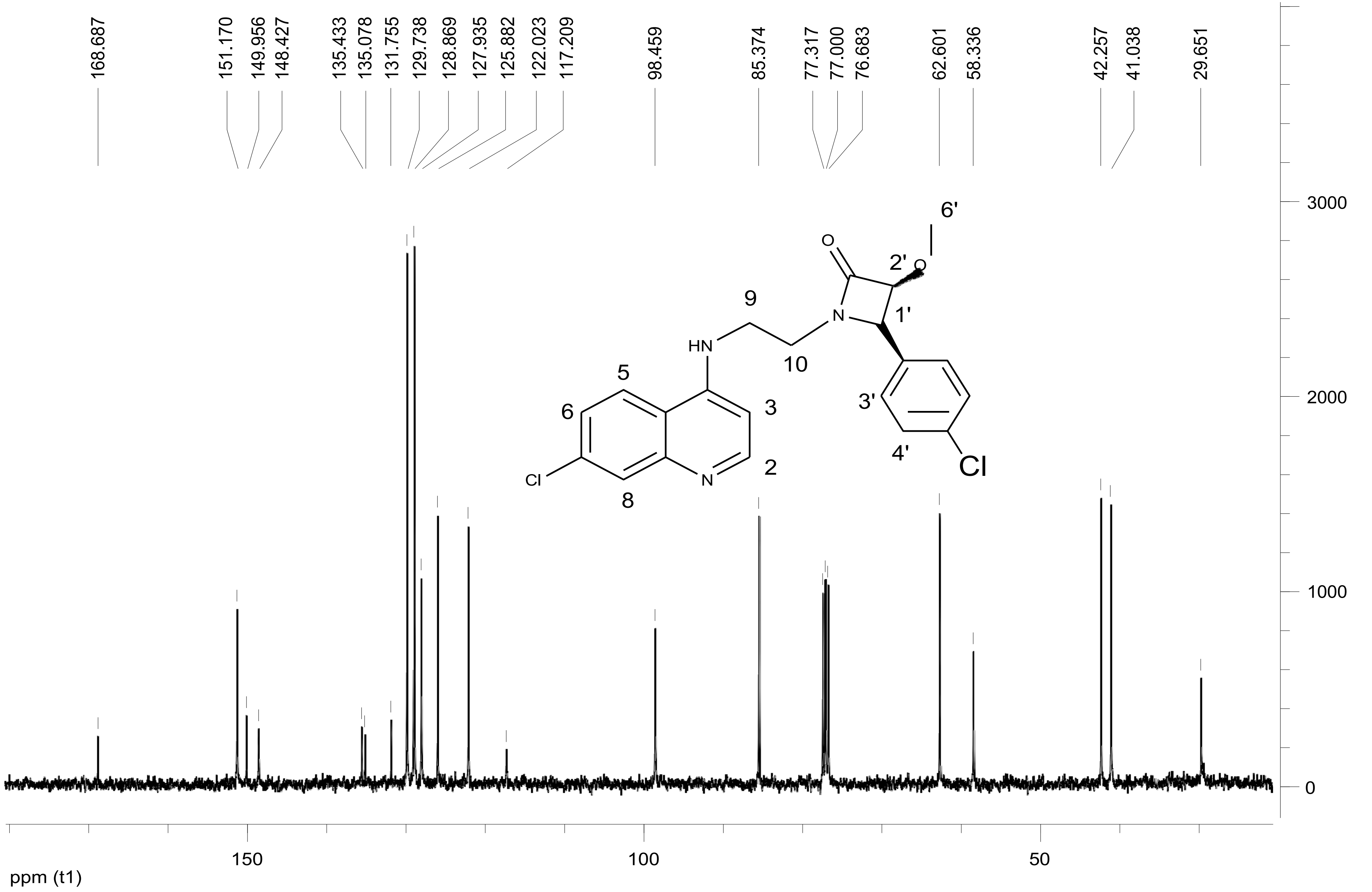
168.687
151.170
149.956
148.427
135.433
135.078
131.755
129.738
128.869
127.935
125.882
122.023
117.209
98.459
85.374
77.317
77.000
76.683
62.601
58.336
42.257
41.038
29.651
3000
2000
1000
0
150
100
50
ppm (t1)
6'
O
2'
O
9
N
1'
HN
10
5
3'
6
3
4'
Cl
2
Cl
N
8

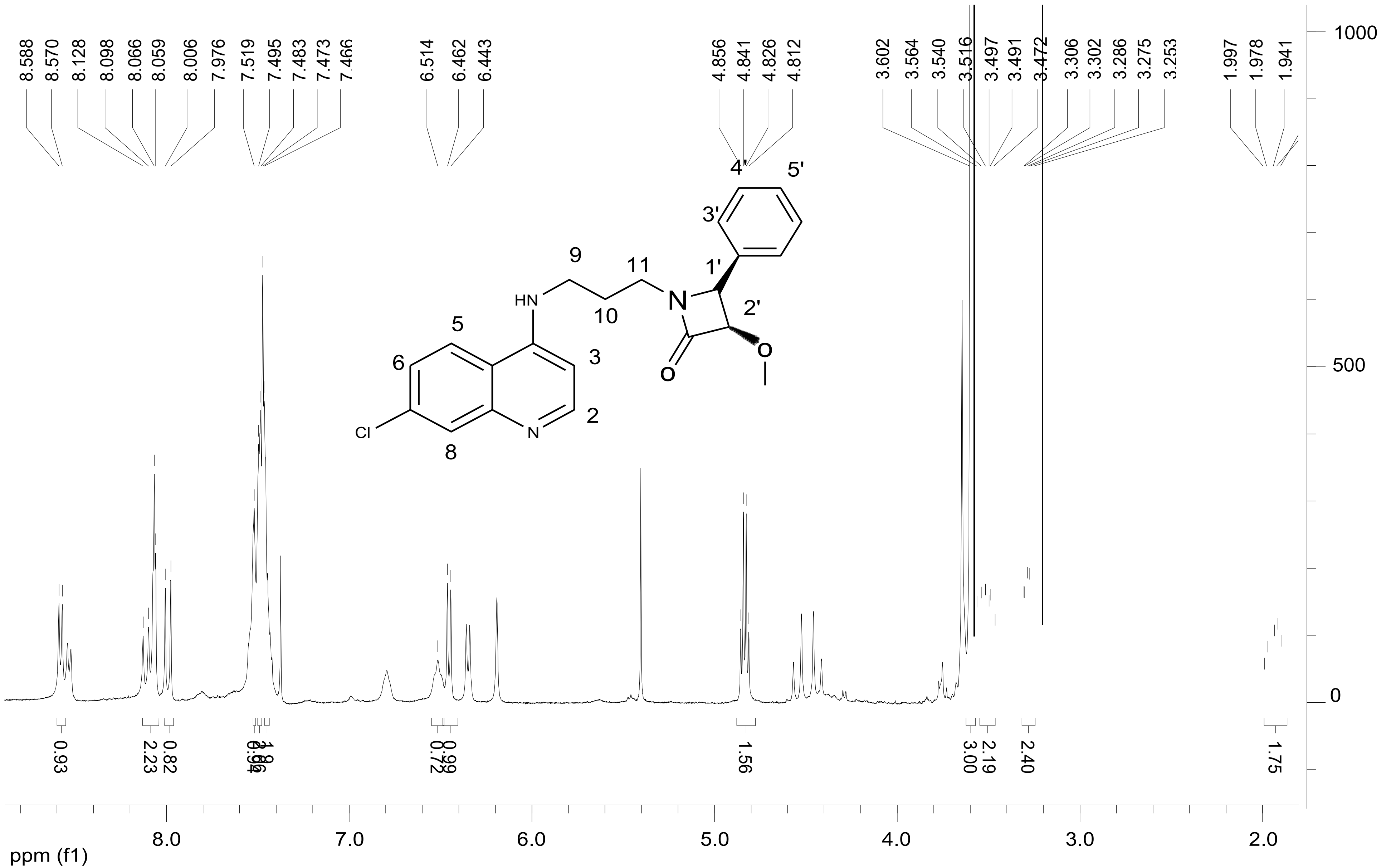

8.588
8.570
8.128
8.098
8.066
8.059
8.006
7.976
7.519
7.495
7.483
7.473
7.466
6.514
6.462
6.443
4.856
4.841
4.826
4.812
3.602
3.564
3.540
3.516
3.497
3.491
3.472
3.306
3.302
3.286
3.275
3.253
1.997
1.978
1.941
1000
500
0
0.93
2.23
0.82
0.72
0.99
1.56
3.00
2.19
2.40
1.75
8.0
7.0
6.0
5.0
4.0
3.0
2.0
ppm (f1)
HN
N
O
Cl
2
3
5
6
8
9
10
11
1'
2'
3'
4'
5'

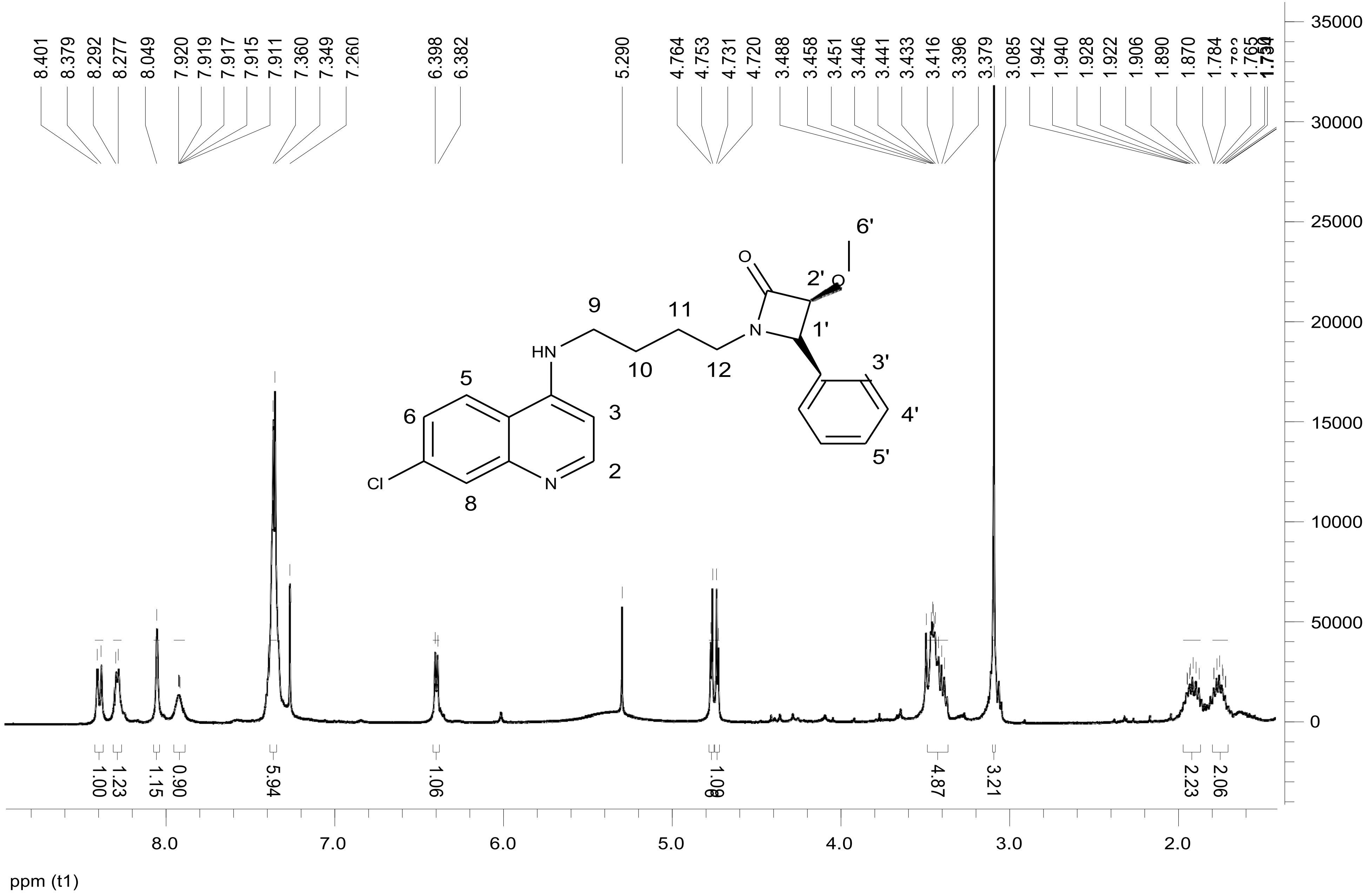
8.401
8.379
8.292
8.277
8.049
7.920
7.919
7.917
7.915
7.911
7.360
7.349
7.260
6.398
6.382
5.290
4.764
4.753
4.731
4.720
3.488
3.458
3.451
3.446
3.441
3.433
3.416
3.396
3.379
3.085
1.942
1.940
1.928
1.922
1.906
1.890
1.870
1.784
1.765
6'
O
2'
O
9
11
N
1'
HN
10
12
3'
5
3
6
4'
2
Cl
N
5'
8
35000
30000
25000
20000
15000
10000
50000
0
1.00
1.23
1.15
0.90
5.94
1.06
1.08
4.87
3.21
2.23
2.06
8.0
7.0
6.0
5.0
4.0
3.0
2.0
ppm (t1)

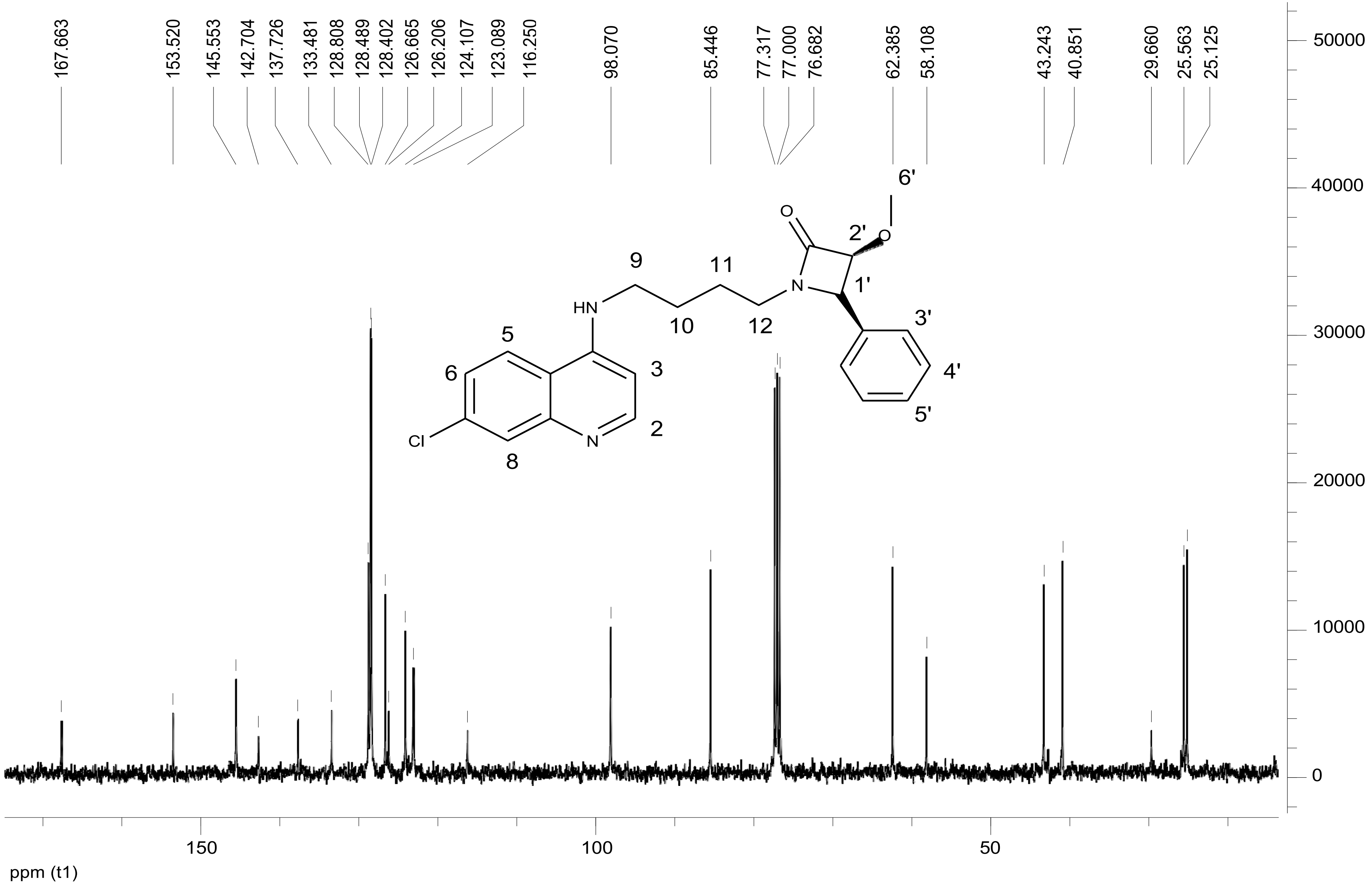

167.663
153.520
145.553
142.704
137.726
133.481
128.808
128.489
128.402
126.665
126.206
124.107
123.089
116.250
98.070
85.446
77.317
77.000
76.682
62.385
58.108
43.243
40.851
29.660
25.563
25.125
50000
40000
30000
20000
10000
0
150
100
50
ppm (t1)
6'
O
2'
O
9
11
HN
N
1'
10
12
3'
5
3
4'
6
Cl
N
2
5'
8

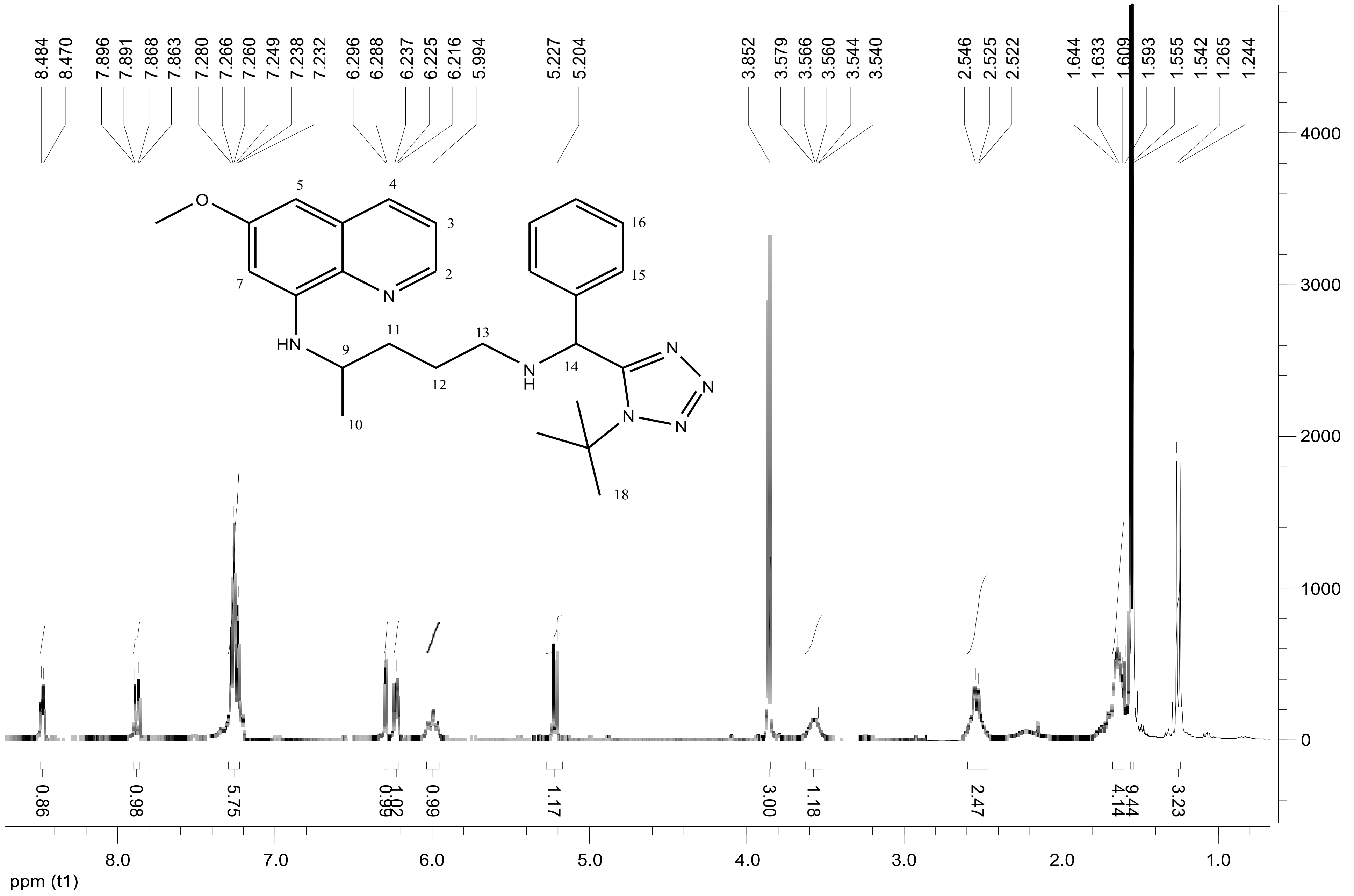

8.484
8.470
7.896
7.891
7.868
7.863
7.280
7.266
7.260
7.249
7.238
7.232
6.296
6.288
6.237
6.225
6.216
5.994
5.227
5.204
3.852
3.579
3.566
3.560
3.544
3.540
2.546
2.525
2.522
1.644
1.633
1.609
1.593
1.555
1.542
1.265
1.244
O
N
HN
N
H
N
N
N
N
2
3
4
5
7
9
10
11
12
13
14
15
16
18
4000
3000
2000
1000
0
0.86
0.98
5.75
0.99
1.02
0.99
1.17
3.00
1.18
2.47
4.14
9.44
3.23
8.0
7.0
6.0
5.0
4.0
3.0
2.0
1.0
ppm (t1)

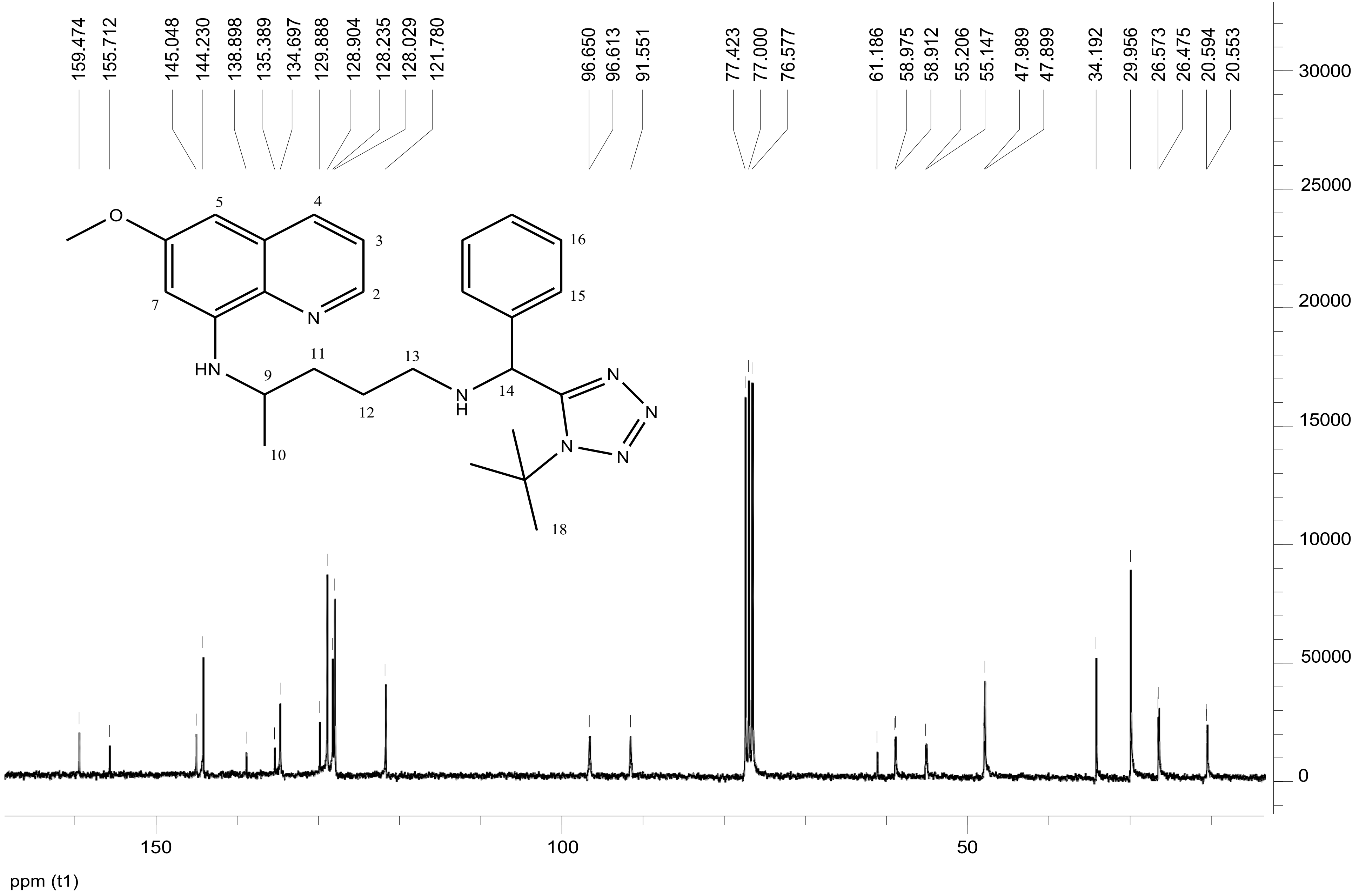

159.474
155.712
145.048
144.230
138.898
135.389
134.697
129.888
128.904
128.235
128.029
121.780
96.650
96.613
91.551
77.423
77.000
76.577
61.186
58.975
58.912
55.206
55.147
47.989
47.899
34.192
29.956
26.573
26.475
20.594
20.553
30000
25000
20000
15000
10000
50000
0
150
100
50
ppm (t1)
O
N
HN
H
N
N
N
N
N
2
3
4
5
7
9
10
11
12
13
14
15
16
18

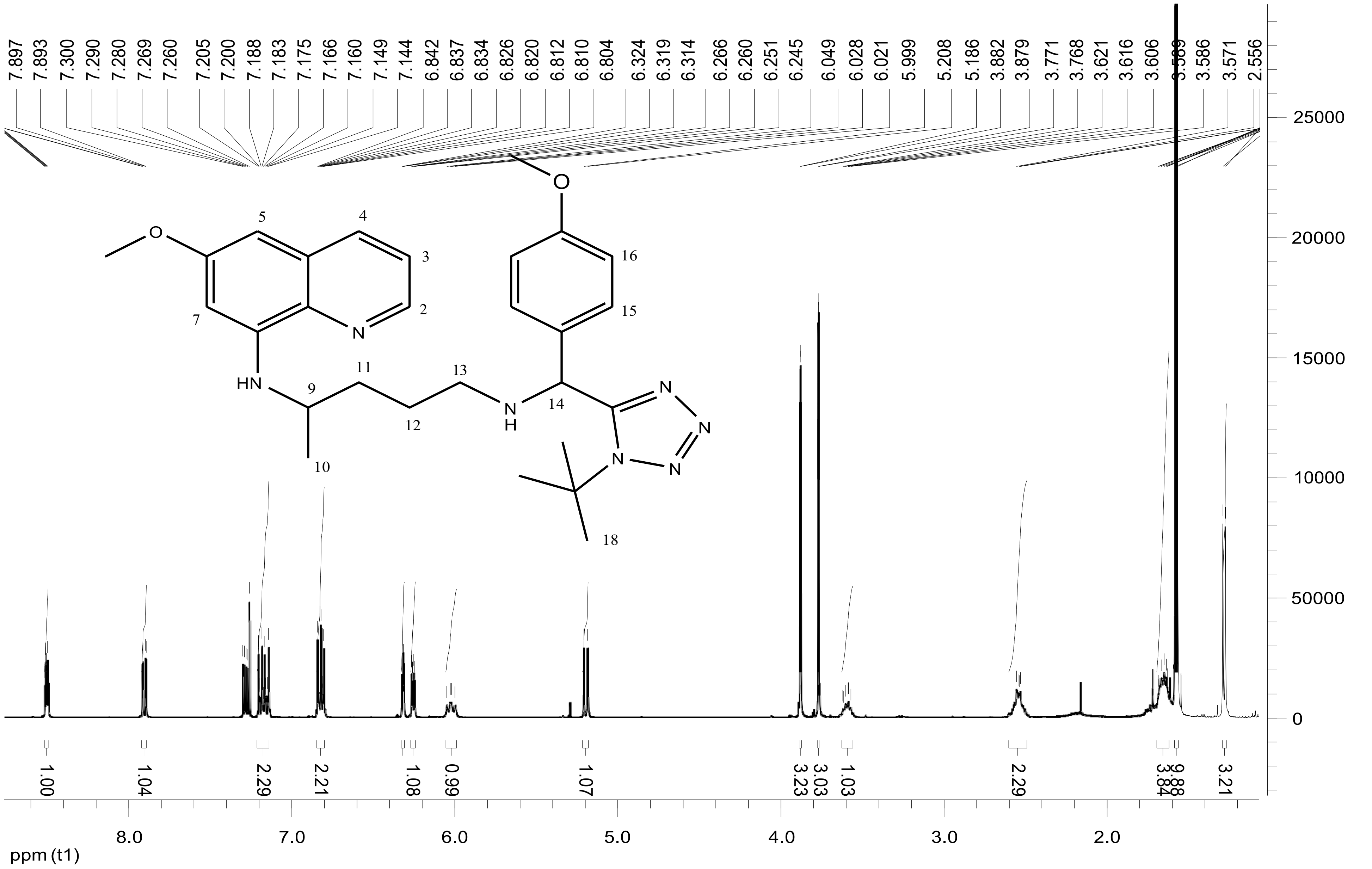
7.897 7.893 7.300 7.290 7.280 7.269 7.260 7.205 7.200 7.188 7.183 7.175 7.166 7.160 7.149 7.144 6.842 6.837 6.834 6.826 6.820 6.812 6.810 6.804 6.324 6.319 6.314 6.266 6.260 6.251 6.245 6.049 6.028 6.021 5.999 5.208 5.186 3.882 3.879 3.771 3.768 3.621 3.616 3.606 3.586 3.571 2.556
HN
NH
N
O
1.00 1.04 2.29 2.21 1.08 0.99 1.07 3.23 3.03 1.03 2.29 3.84 9.88 3.21
8.0 7.0 6.0 5.0 4.0 3.0 2.0
ppm (t1)
25000 20000 15000 10000 50000 0

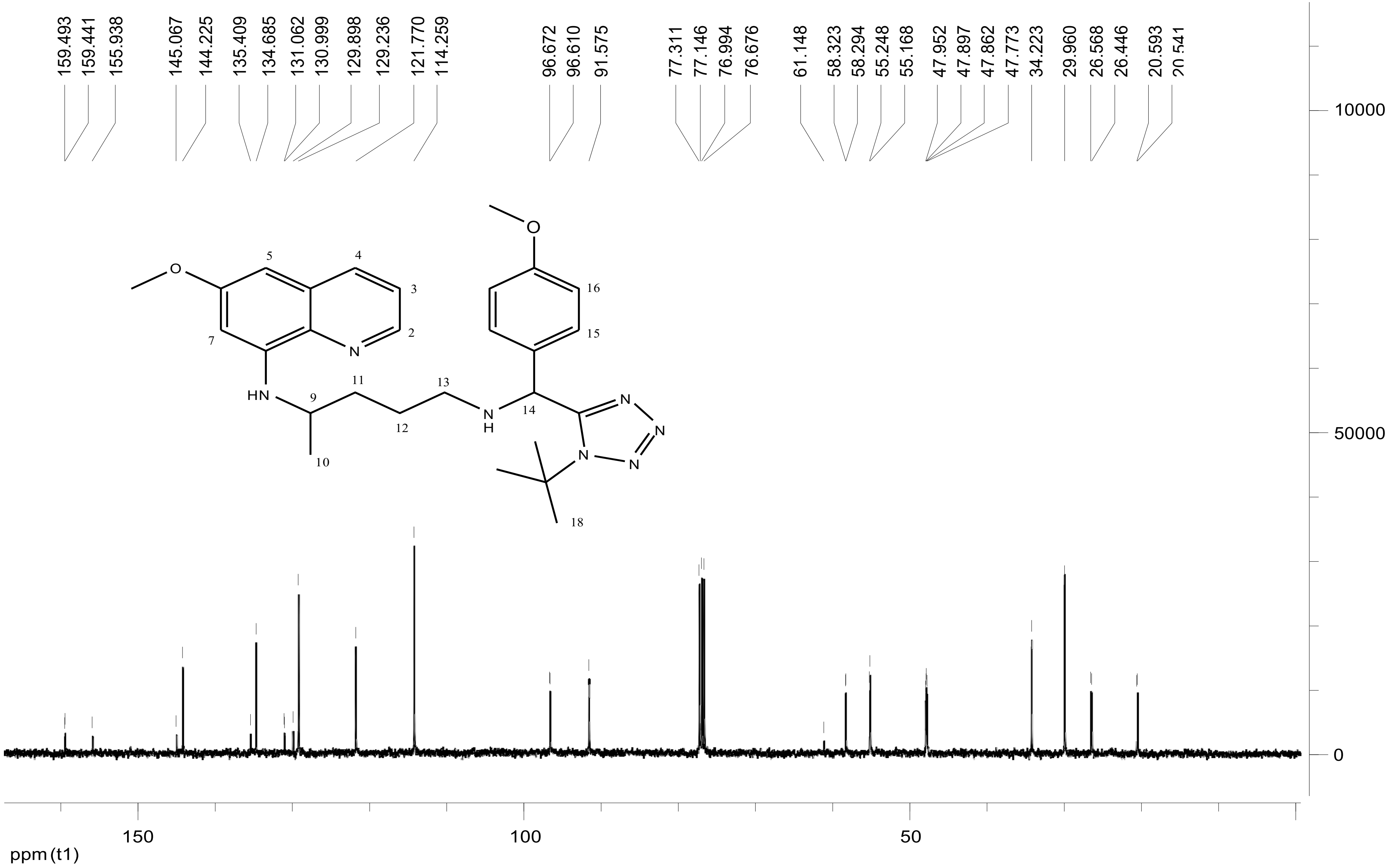

159.493
159.441
155.938
145.067
144.225
135.409
134.685
131.062
130.999
129.898
129.236
121.770
114.259
96.672
96.610
91.575
77.311
77.146
76.994
76.676
61.148
58.323
58.294
55.248
55.168
47.952
47.897
47.862
47.773
34.223
29.960
26.568
26.446
20.593
20.541
10000
50000
0
O
HN
N
H
N
5
4
3
2
7
9
10
11
12
13
14
15
16
18
150
100
50
ppm (t1)

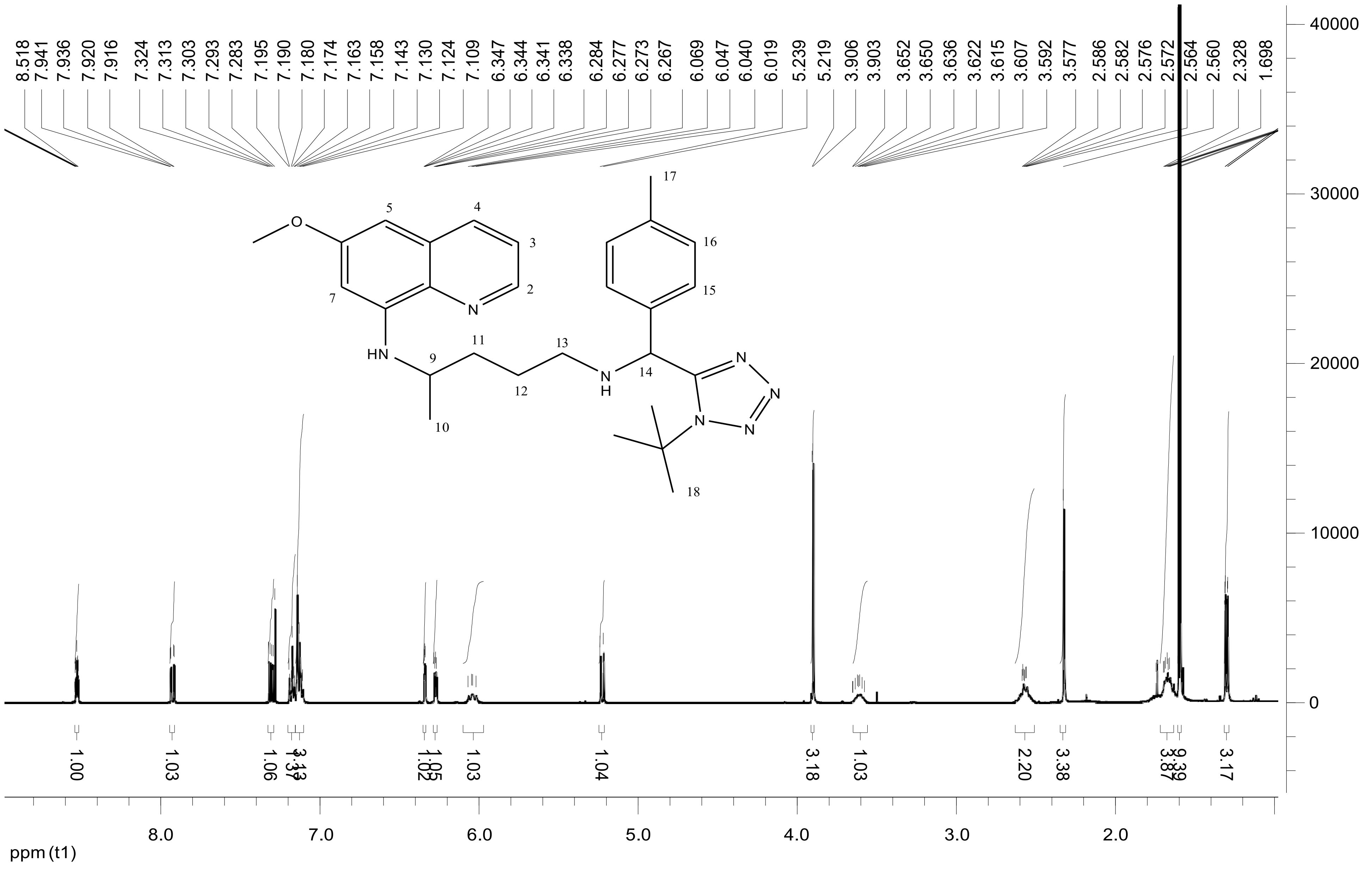

8.518 7.941 7.936 7.920 7.916 7.324 7.313 7.303 7.293 7.283 7.195 7.190 7.180 7.174 7.163 7.158 7.143 7.130 7.124 7.109 6.347 6.344 6.341 6.338 6.284 6.277 6.273 6.267 6.069 6.047 6.040 6.019 5.239 5.219 3.906 3.903 3.652 3.650 3.636 3.622 3.615 3.607 3.592 3.577 2.586 2.582 2.576 2.572 2.564 2.560 2.328 1.698
O
N
HN
N
H
N
N
N
N
1.00
1.03
1.06
1.04
3.18
1.03
2.20
3.38
3.17
8.0
7.0
6.0
5.0
4.0
3.0
2.0
ppm (t1)
40000
30000
20000
10000
0

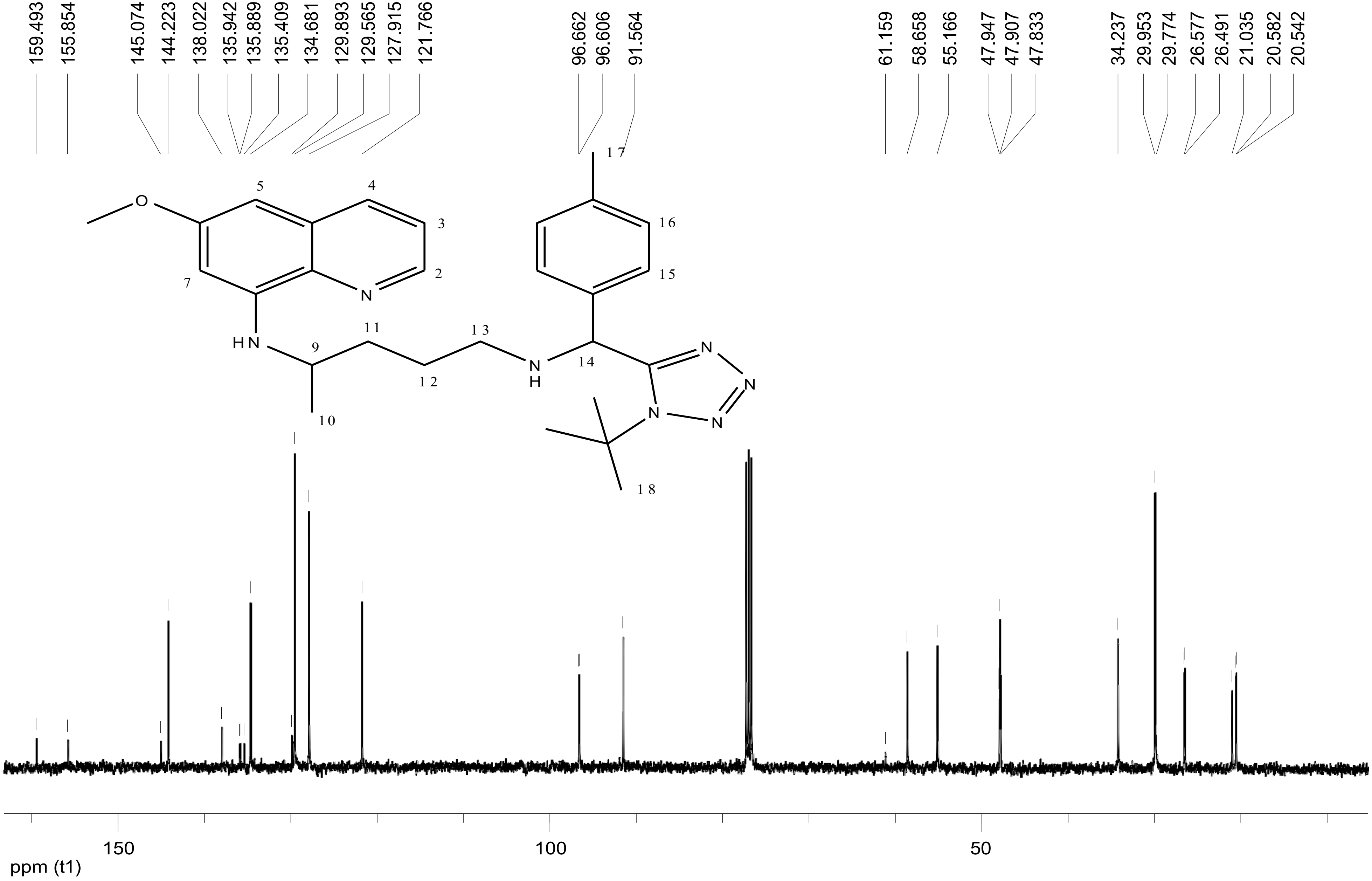

159.493
155.854
145.074
144.223
138.022
135.942
135.889
135.409
134.681
129.893
129.565
127.915
121.766
96.662
96.606
91.564
61.159
58.658
55.166
47.947
47.907
47.833
34.237
29.953
29.774
26.577
26.491
21.035
20.582
20.542
150
100
50
ppm (t1)

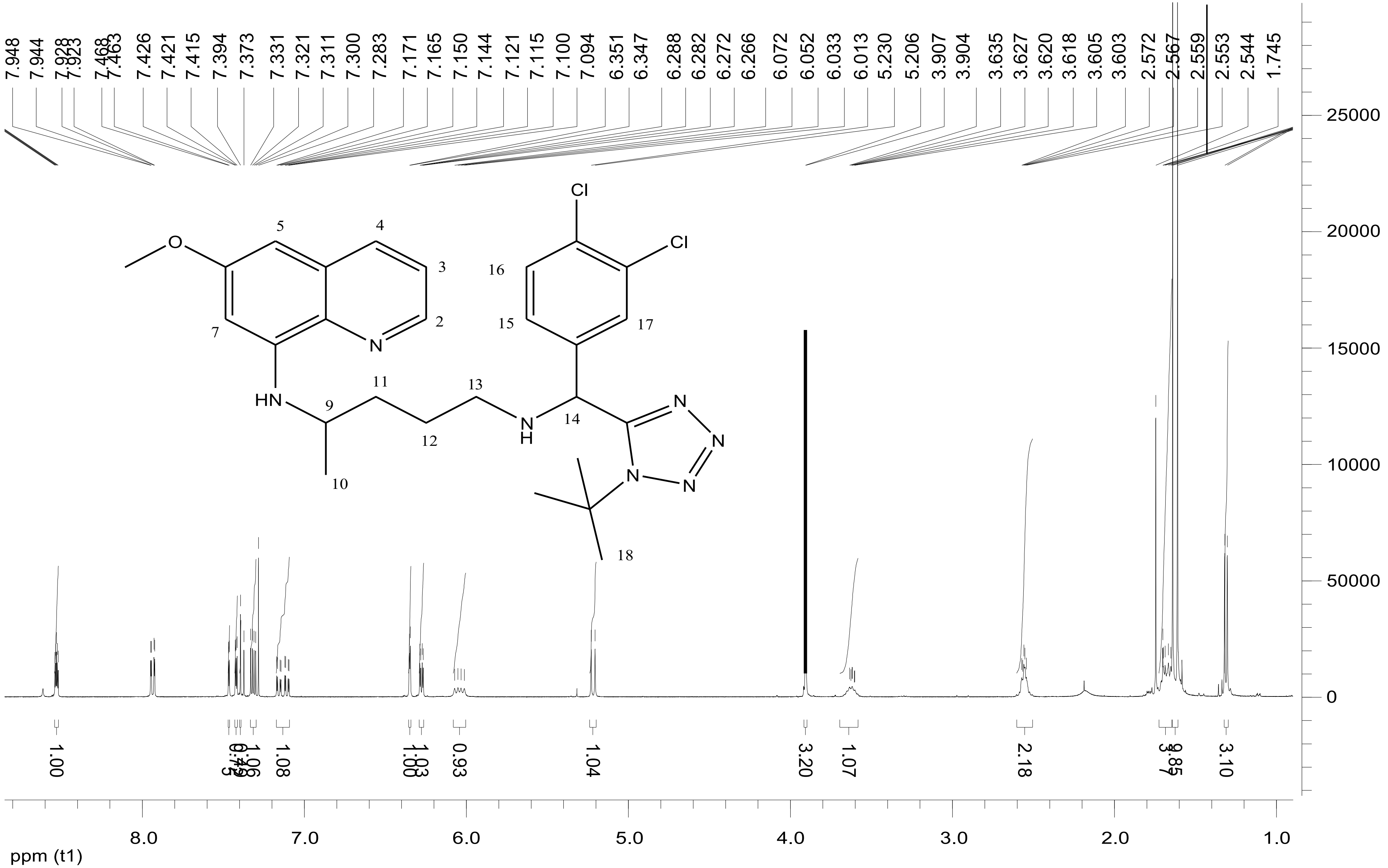

7.948
7.944
7.928
7.923
7.468
7.463
7.426
7.421
7.415
7.394
7.373
7.331
7.321
7.311
7.300
7.283
7.171
7.165
7.150
7.144
7.121
7.115
7.100
7.094
6.351
6.347
6.288
6.282
6.272
6.266
6.072
6.052
6.033
6.013
5.230
5.206
3.907
3.904
3.635
3.627
3.620
3.618
3.605
3.603
2.572
2.567
2.559
2.553
2.544
1.745
Cl
Cl
O
N
HN
N
H
N
N
N
N
1
2
3
4
5
7
9
10
11
12
13
14
15
16
17
18
25000
20000
15000
10000
50000
0
1.00
1.08
1.00
1.03
0.93
1.04
3.20
1.07
2.18
3.10
8.0
7.0
6.0
5.0
4.0
3.0
2.0
1.0
ppm (t1)

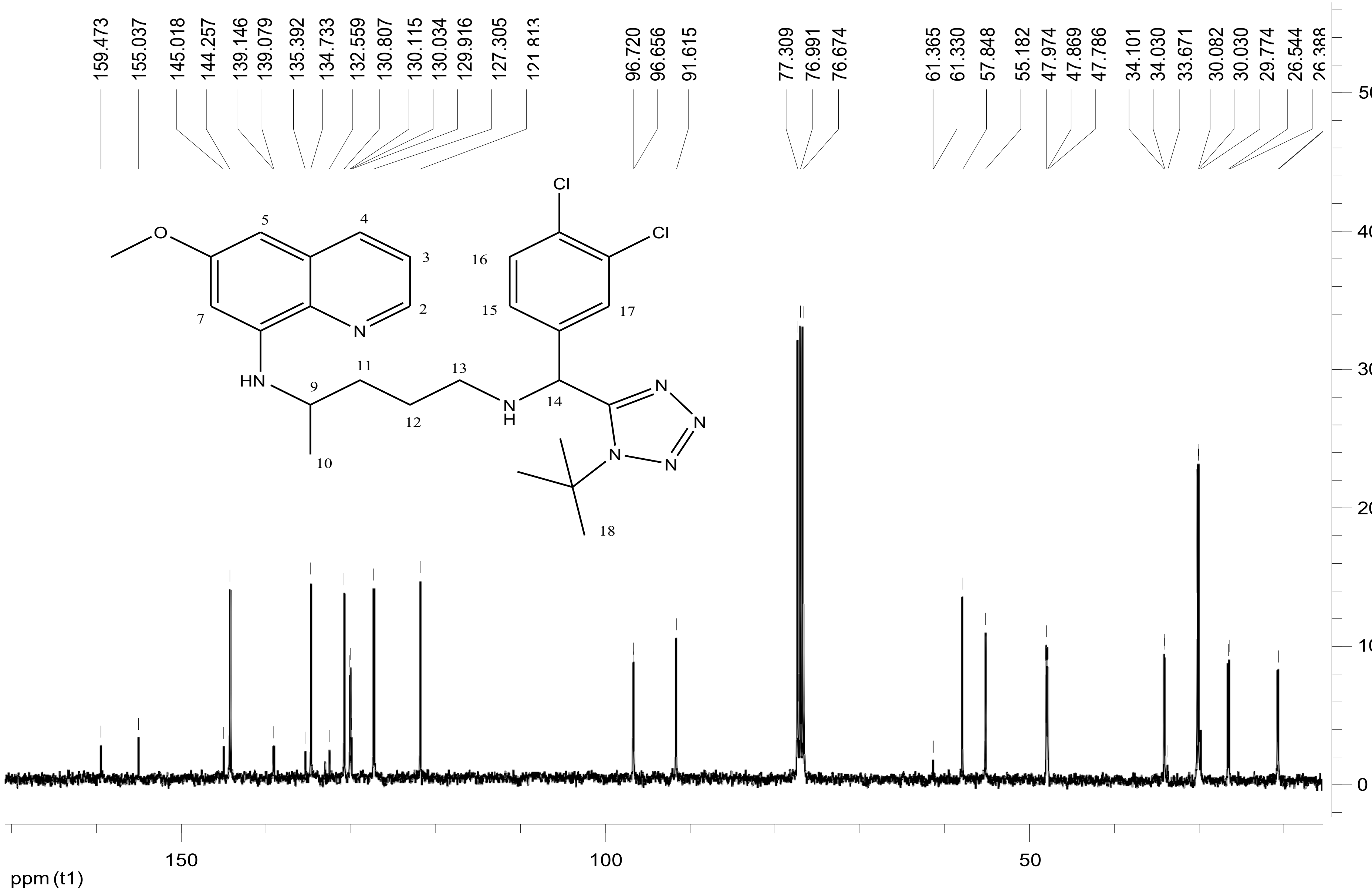

159.473
155.037
145.018
144.257
139.146
139.079
135.392
134.733
132.559
130.807
130.115
130.034
129.916
127.305
96.720
96.656
91.615
77.309
76.991
76.674
61.365
61.330
57.848
55.182
47.974
47.869
47.786
34.101
34.030
33.671
30.082
30.030
29.774
26.544
26.388
50000
40000
30000
20000
10000
0
150
100
50
ppm (t1)
O
N
HN
H
N
Cl
Cl
N
N
N
N
2
3
4
5
7
9
10
11
12
13
14
15
16
17
18

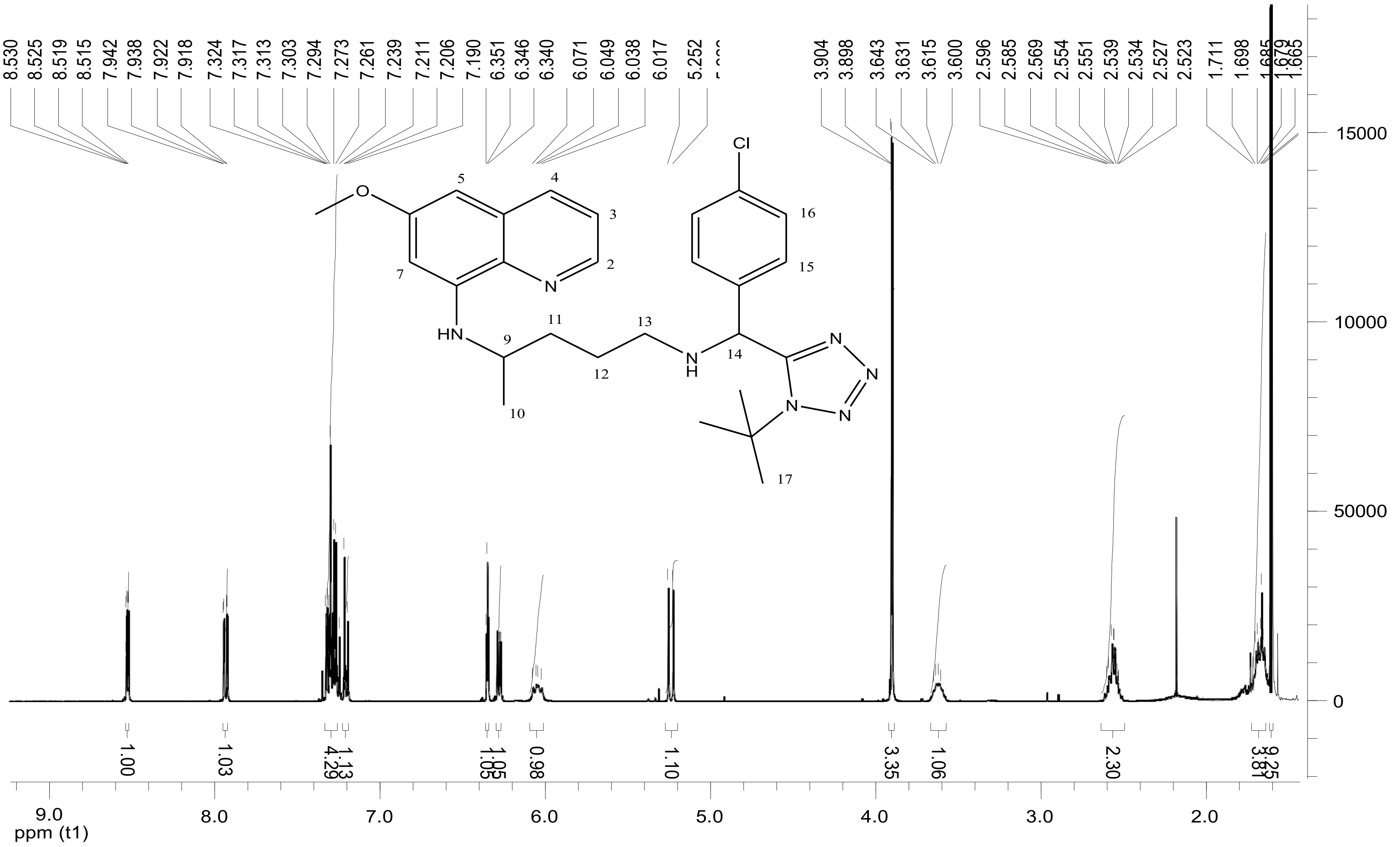

8.530 8.525 8.519 8.515 7.942 7.938 7.922 7.918 7.324 7.317 7.313 7.303 7.294 7.273 7.261 7.239 7.211 7.206 7.190 6.351 6.346 6.340 6.071 6.049 6.038 6.017 5.252
3.904 3.898 3.643 3.631 3.615 3.600 2.596 2.585 2.569 2.554 2.551 2.539 2.534 2.527 2.523 1.711 1.698
Cl
HN
N
H
N
N
N
N
O
1.00 1.03 4.29 1.13 1.05 1.05 0.98 1.10 3.35 1.06 2.30 3.81 9.25
15000
10000
50000
0
9.0 8.0 7.0 6.0 5.0 4.0 3.0 2.0
ppm (t1)

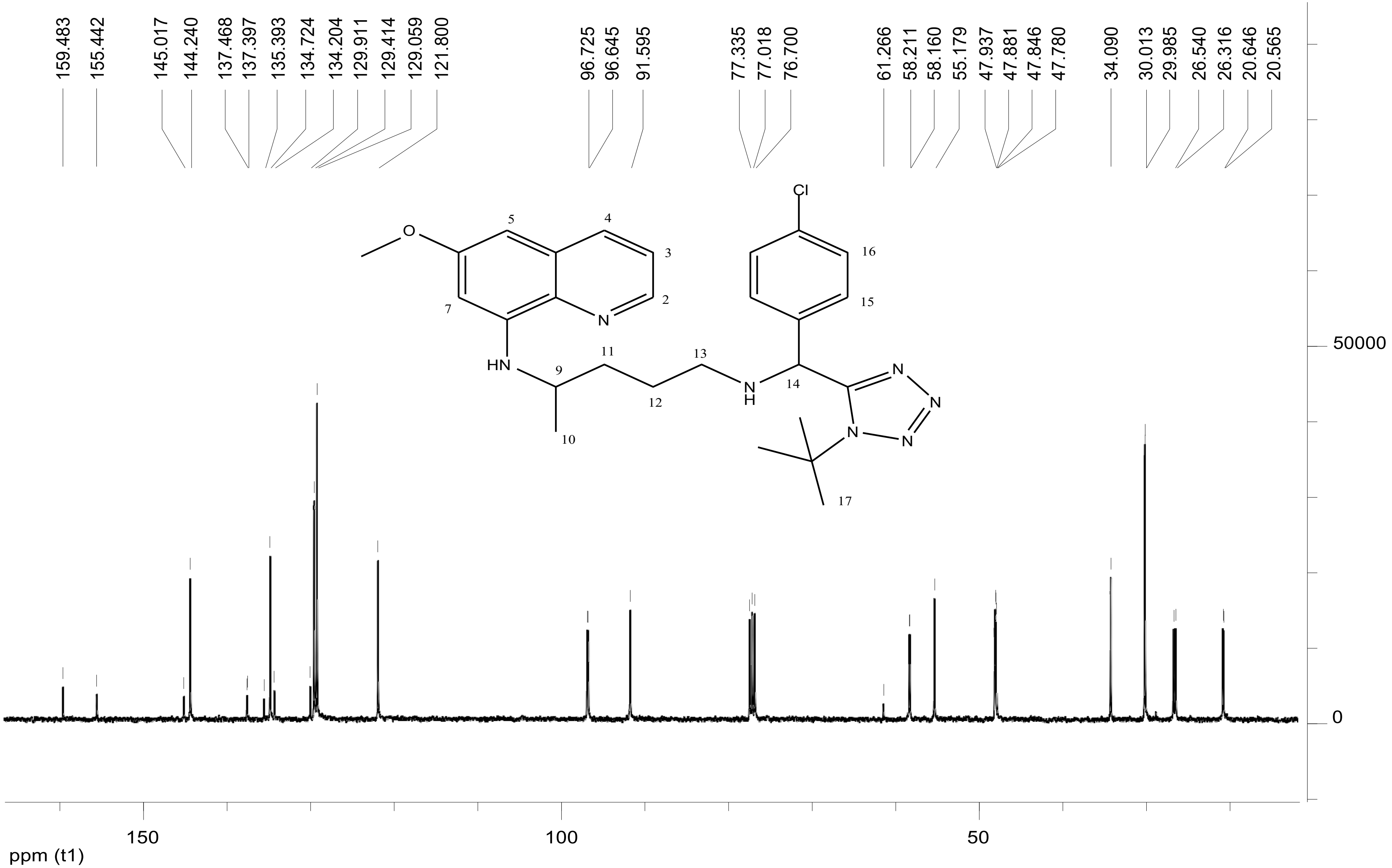

159.483
155.442
145.017
144.240
137.468
137.397
135.393
134.724
134.204
129.911
129.414
129.059
121.800
96.725
96.645
91.595
77.335
77.018
76.700
61.266
58.211
58.160
55.179
47.937
47.881
47.846
47.780
34.090
30.013
29.985
26.540
26.316
20.646
20.565
Cl
O
5
4
3
2
7
N
16
15
HN
9
11
13
N
H
14
N
N
N
N
12
10
17
50000
0
150
100
50
ppm (t1)
